# Defining the Threshold IL-2 Signal Required for Induction of Selective Treg Cell Responses Using Engineered IL-2 Muteins

**DOI:** 10.3389/fimmu.2020.01106

**Published:** 2020-06-05

**Authors:** Aazam Ghelani, Darren Bates, Kip Conner, Min-Zu Wu, Jiamiao Lu, Yi-Ling Hu, Chi-Ming Li, Ashutosh Chaudhry, Sue J. Sohn

**Affiliations:** ^1^Amgen Research, Amgen Inc, South San Francisco, CA, United States; ^2^Amgen Research, Amgen Inc, Thousand Oaks, CA, United States

**Keywords:** IL-2, mutein, regulatory T, Treg, tolerance, inflammation, autoimmunity

## Abstract

Among all T and NK cell subsets, regulatory T (Treg) cells typically respond to the lowest concentrations of IL-2 due to elevated surface expression of the IL-2R alpha chain (IL2RA; CD25) and the high affinity IL-2 receptor (IL-2R) complex. This enhanced sensitivity forms the basis for low-dose (LD) IL-2 therapy for the treatment of inflammatory diseases, where efficacy correlates with increased Treg cell number and expression of functional markers. Despite strong preclinical support for this approach, moderate and variable clinical efficacy has raised concerns that adequate Treg selectivity still cannot be achieved with LD IL-2, and/or that doses are too low to stimulate effective Treg-mediated suppression within tissues. This has prompted development of IL-2 variants with greater Treg selectivity, achieved through attenuated affinity for the signaling chains of the IL-2R complex (IL2RB or CD122 and IL2RG or CD132) and, consequently, greater reliance on high CD25 levels for full receptor binding and signaling. While certain IL-2 variants have advanced to the clinic, it remains unknown if the full range of IL-2R signaling potency and Treg-selectivity observed with low concentrations of wildtype IL-2 can be sufficiently recapitulated with attenuated IL-2 muteins at high concentrations. Using a panel of engineered IL-2 muteins, we investigated how a range of IL-2R signaling intensity, benchmarked by the degree of STAT5 phosphorylation, relates to biologically relevant Treg cell responses such as proliferation, lineage and phenotypic marker expression, and suppressor function. Our results demonstrate that a surprisingly wide dynamic range of IL-2R signaling intensity leads to productive biological responses in Treg cells, with negligible STAT5 phosphorylation associating with nearly complete downstream effects such as Treg proliferation and suppressor activity. Furthermore, we show with both *in vitro* and humanized mouse *in vivo* systems that different biological responses in Treg cells require different minimal IL-2R signaling thresholds. Our findings suggest that more than minimal IL-2R signaling, beyond that capable of driving Treg cell proliferation, may be required to fully enhance Treg cell stability and suppressor function *in vivo*.

## Introduction

Produced primarily by activated T cells, IL-2 influences critical aspects of the immune response and homeostasis. IL-2 serves dual opposing functions; it potently amplifies proliferative responses of effector T (Teff) and natural killer (NK) cells, while regulating immune homeostasis by driving regulatory T (Treg) cell proliferation, differentiation, and function [review by Abbas et al. (1)]; and both axes have been leveraged to treat human diseases. In cancer patients, high-dose IL-2 therapy enhances Teff and NK cell mediated tumor cell killing (2). More recently, low-dose IL-2 therapy has been tested in inflammatory and autoimmune diseases where Treg expansion and increased expression of functional markers have been correlated with disease improvement (3–7). The efficacy of the low-dose IL-2 therapy has been attributed to the fact that Treg cells exhibit exquisite sensitivity to IL-2 (8) compared to other cell types (9–11), and thus, Treg cells preferentially respond when IL-2 availability is limited. However, the therapeutic dose range is narrow, as doses that induce more robust Treg cell responses simultaneously drive Teff and NK cells activity, which can reduce efficacy or lead to disease exacerbation and/or toxicity (3, 4, 7, 12).

Efforts to widen the therapeutic window of low-dose IL-2 therapy in recent years have focused around molecular engineering to increase the selectivity of IL-2 on Treg vs. Teff and NK cells. Predominantly, the engineered versions of IL-2 with increased selectivity for Treg cells possess attenuated binding and/or activity toward the IL2RB chain or IL2RG chain (13–16) which increases dependence on CD25 for generating stable interactions with the IL2RBG signaling chains, thereby enhancing selectivity for cells that express higher levels of CD25, such as Treg cells. Weaker activity also restrains the undesirable effects on Teff and NK cells over a wider concentration range (17). Similarly, IL-2:IL-2 Ab complexes that generate an attenuated IL-2 signal have been reported to demonstrate increased Treg cell selectivity (18).

The critical role of IL-2 in regulating Treg cell number and function is supported by human genetic studies and mouse models that lack various components of the IL-2 and IL-2R pathway. For example, the phenotype of mice lacking the expression of IL-2, or IL2RA or IL2RB chain (19, 20), or the downstream transcription factor STAT5 (21), recapitulates a wide range of the defects observed in the Foxp3 loss-of-function *scurfy* strain and Foxp3-deficient mice that lack functional Treg cells (22–25). In the absence of the IL-2 signal, Treg cell numbers are reduced (but not completely absent), they express reduced levels of Foxp3 and other phenotypic and activation markers, and they lose their suppressor function, which result in a fatal lymphoproliferative and autoimmune disease. In people, IL2RA deficiency (26–28) or STAT5B gene mutations (29) has been correlated with diseases that manifest aspects of autoimmunity, and additionally, allelic variants of the IL-2 or IL-2R or downstream genes have been identified in association with increased risks for autoimmune inflammatory diseases [review in Abbas et al. and Humrich et al. (1, 30)]. In further support, reduced IL-2 production or IL-2R signaling has been observed in human patients with autoimmune diseases such as type 1 diabetes (T1D) [review by Long et al. and Hull et al. (31, 32)] and systemic lupus erythematosus (SLE) (30). Low-dose IL-2 treatment is aimed to remedy such a proximal deficit and to further boost the IL-2-dependent effects on Treg cells, the primary outcome being the expansion in number and possibly an enhancement of their suppressive function.

As mice that completely lack the expression of IL-2 or its receptor still develop Treg cells (20, 33, 34), it is thought that cytokines other than IL-2 (e.g., IL-15) that can activate STAT5 can compensate and promote survival and expansion during early Treg differentiation (21), or that certain aspects of early Treg cell differentiation do not require IL-2. The fact that fatal disease develops in these mice suggests that, even though present, these Treg cells do not behave as effective tolerance mediators. Furthermore, ablation of IL-2R selectively in mature Treg cells results in a similar fatal lymphoproliferative inflammatory disease observed in mice that completely lack Treg cells (33, 34), indicating that continuous IL-2 signal is required to maintain mature Treg function *in vivo*. Similar disparity has been observed in human patients, where Foxp3+ Treg cells exist but are insufficient at controlling pathogenic inflammation (35). In some cases, Treg cells from these patients display reduced sensitivity to IL-2 stimulation (36–38), suggesting that Treg cells lose their functional capacity in the absence of a certain threshold level of IL-2 signaling. These data suggest that biological responses of Treg cells are sensitive to different levels of IL-2 signal. For example, one can hypothesize that only minimal IL-2R signals are required to maintain a normal Treg population through modest proliferative and survival signals, while more robust IL-2R signals are required to support maximal suppressor function(s) and maintain Treg stability in inflammatory settings.

With an interest in engineering an attenuated IL-2 that would preferentially bolster Treg cell number as well as function in clinical settings, we hypothesized that variable IL-2 signaling potency would contribute differentially to a number of key biological responses in human Treg cells. We generated a panel of engineered IL-2 molecules with mutations (referred to as IL-2 muteins) that impact binding to IL2RB and/or CD25 and evaluated how Treg and non-Treg cells responded to attenuated IL-2 signal. Using STAT5 activation as a quantifier of the proximal IL-2R signal, we compared Treg vs. non-Treg cell responses to assess relative selectivity of these molecules and further attempted to define the threshold IL-2 signal required to trigger meaningful biological responses in Treg and non-Treg cells. Our data show that human Treg cells can tolerate a significant degree of attenuation in IL-2 signal for certain biological responses. Nonetheless, the key Treg cell responses that collectively contribute toward their effectiveness as an immune suppressor were quantitatively dependent on IL-2 signal, indicating that IL-2 is a requisite driver of mature human Treg cell function. In contrast, non-Treg T cells do not tolerate attenuated IL-2 signals, providing an explanation for how attenuated IL-2 muteins increase Treg-to-non-Treg selectivity. In addition, our results suggest that Treg cell proliferation, activation marker expression, and suppressor function are sensitive to different threshold levels of IL-2R signaling, indicating that the requirements for IL-2 signal in Treg cells are heterogeneous.

## Results

### Structure-Based Design of IL-2 Muteins With Attenuated Interactions to CD25 and/or IL2RB

**Figure 1:**
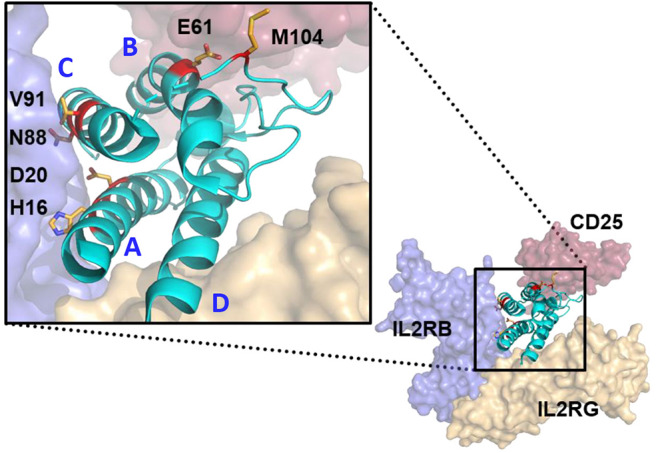
Structure modeling of huIL-2:huIL-2 trimeric receptor complex and the amino acid residues mutated to attenuate the interaction with the various receptor chains. Model was generated from Pymol based on 2B5I pdb of the IL-2 co-crystal structure. CD25, IL2RB, and IL2RG are displayed as red, blue, and gold, respectively. IL-2 ligand is represented in Cyan with residues mutated in the study with side chains displayed and colored in red. Helices (A–D) are indicated in blue. IL-2 mutations that attenuate IL2RB are surface exposed residues which directly contact IL2RB.

To explore the impact of weakened interaction of IL-2 with its receptor, mutations were designed at three interfaces. The first approach was to attenuate interactions with IL2RB directly by altering IL-2 at the core interface in the IL-2 A and C helices (Figure 1). The second was to slightly attenuate binding to CD25 at the interface edge. The third was focused on removal of a highly surface exposed methionine that shows no contact to any of the three receptors to improve manufacturability. Combinations of mutations were made between different IL2RB attenuation mutations to further decrease potency, between IL2RB and CD25 contact residues to mix receptor attenuation, and with IL2RB and methionine mutations for attenuation and manufacturability. Structural analysis of the IL-2 cytokine-receptor quaternary complex crystal structure (PDB 2B5I) was performed using Pymol. The structural analysis along with data from single mutation analysis from previous studies (39) were the basis for the following designs. The helices A and C, which form the IL-2 interface with IL2RB, were mutated at 4 positions as single or double combination substitutions. The histidine at position 16 (H16) was mutated to longer charged residues E or R to introduce steric repulsion due to a lack of space available in the histidine pocket. The aspartate at position 20 (D20) was mutated to disrupt electrostatic and Van der Waals interactions by substitution with alanine, or increase steric repulsion by mutation to tryptophan, respectively. The loss of binding was expected to be stronger with the D20W mutation than the D20A, therefore double attenuation mutations were not made with D20W. Weakening of the IL-2:IL2RB interface at position N88 to either D (13) or K substitutions disrupts a bidentate hydrogen bonding pair. Mutation of the valine at position 91 (V91) to small polar serine, positively charge lysine, or negatively charged aspartate was designed to create a weak or moderate attenuation as it is adjacent to the core interface. Mutations predicted or previously observed to impart minor impact were combined to produce stronger attenuation in variants such as D20A/H16E, D20A/H16R, or V91K/D20A. A single mutation of E61Q was tested to partially disrupt the CD25 interface by reduction of an electrostatic interaction with lysine from CD25. The mutation of M104 to T, L, or V was designed to improve manufacturability by removing an oxidation site. The mutated IL-2 variants were fused to the Fc portion of human IgG1 containing an N297G mutation, which abrogates the Fc effector functions. The resulting molecule was bivalent for IL-2 which could result in avidity interactions with IL-2R.

### The Mutein Panel Induces a Broad Spectrum of IL-2 Activity Measured by STAT5 Activation

IL-2 induces an intracellular signal via the heterodimeric receptor complex composed of IL2RB and IL2RG [review by Taniguchi and Minami (40, 41)]. CD25 can bind to IL-2 at low affinity by itself but does not induce a signal. However, inclusion of CD25 converts the intermediate affinity IL2RB:IL2RG heterodimeric receptor complex to a high affinity heterotrimeric receptor complex. Binding of IL-2 to the receptor activates JAK1 and JAK3 tyrosine kinases that are associated with the cytoplasmic tails of IL2RB and IL2RG chains, respectively. Activated JAK kinases phosphorylate tyrosine residues within the cytoplasmic tails of IL2RB and IL2RG, which serve as docking sites for downstream signaling molecules. Recruitment and activation of STAT5 is considered a critical event in IL-2 signaling (42, 43). Therefore, we evaluated relative potencies of our IL-2 muteins using STAT5 activation as the receptor proximal readout, measured by phospho STAT5 (pSTAT5) in a flow cytometry-based assay.

**Figure 2:**
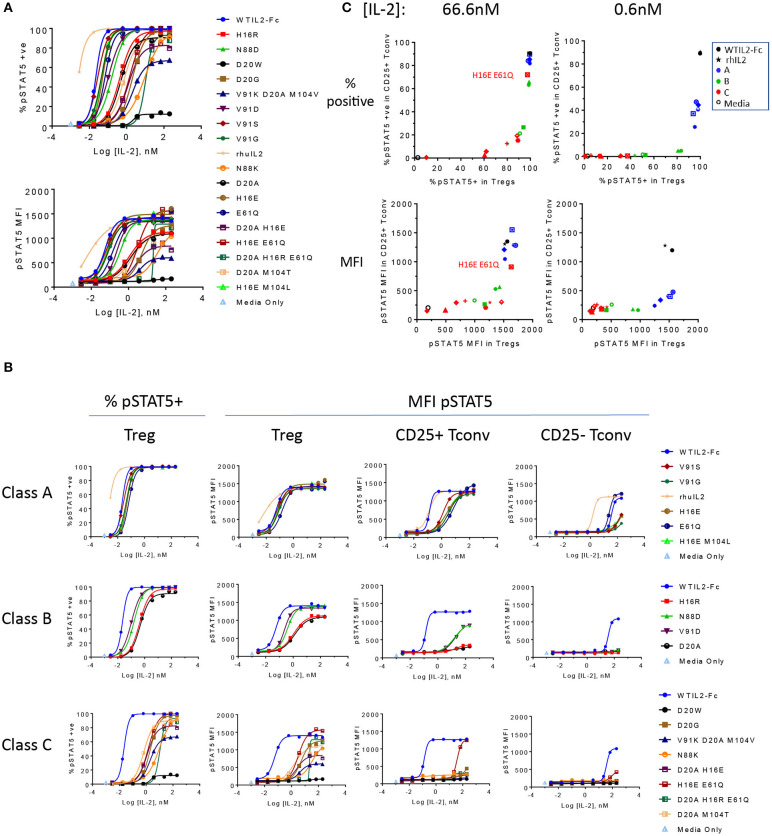
The engineered IL-2 mutein panel induces a broad spectrum of pSTAT5 response. **(A)** Activity of the individual IL-2 muteins is represented by phospho STAT5 (pSTAT5) signal at increasing concentrations of IL-2 muteins in human whole blood cell assay. pSTAT5 responses are shown as both the percent (%) pSTAT5-positive cells in (top panel) and pSTAT5 MFI of (bottom panel) CD25hi Foxp3+ gated Treg cells (gating of an example plot shown in Supplementary Figure 1). Shown are representative plots of data from three donors. **(B)** The same pSTAT5 data shown in **(A)** are represented according to the potency-based classification to illustrate the impact of attenuation on various cell populations. Both the percent positive and pSTAT5 MFI for Treg gated populations are shown. In addition, pSTAT5 MFI data are shown for CD25+ Tconv, and CD25– Tconv cells. **(C)** The activity of the muteins on CD25+ Tconv cells vs. Treg cells are shown in 2D plots. Percent pSTAT5-positive and pSTAT5 MFI readouts at mutein concentrations 66.6 and 0.6 nM are shown, color coded to represent wildtype IL-2 as the Fc fusion (WTIL2-Fc, black circle), free recombinant cytokine (rhIL2, black star), class A (blue), B (green), C (red) muteins, and media only control (black open circle).

Since Treg cells constitutively express high levels of the high affinity IL-2R (8, 9) and are considered the most sensitive responders to IL-2 (9, 44, 45), we rank-ordered the muteins based on their activity on Treg cells (gating shown in Supplementary Figure 1A) measured by pSTAT5. As shown in Figure 2A, the dose response curves of the muteins in our panel show a wide range of activities, with varying degrees of attenuation compared to wild type IL-2. We compared the calculated EC50 values based on both the percent pSTAT5-positive cells and the mean pSTAT5 levels (Supplementary Figure 1B) indicated by the median fluorescence intensity (MFI) and determined that the rank orders of the mutein potencies were similar. To better understand how the attenuation impacted downstream biological responses, and to simplify the comparison amongst different muteins, we grouped them into three classes using arbitrary cutoff points established based on their activity relative to wildtype IL-2 (Table 1).

**Table 1:** Mutein classification based on the pSTAT5 MFI EC50 values.

**Class**	**Muteins**	**AVG EC50 (nM), *n* = 3**	**STDEV EC50**	**AVG dMFI at 66.7 nM, *n* = 3**
A	rhIL2	0.008	0.0097	1455.3
	WT	0.057	0.0054	1501.3
	H16E M104L	0.062	0.0257	1225.3
	H16E	0.067	0.0244	1277.7
	V91S	0.089	0.0393	1154.7
	E61Q	0.117	0.0300	1680.3
	V91G	0.153	0.0768	1055.0
B	V91D (A/B)	0.367	0.1415	1000.0
	N88D (A/B)	0.418	0.0564	1032.7
	H16R	1.168	0.1601	860.3
	D20A	1.205	0.8115	939.0
C	D20A H16E	1.596	0.8670	697.7
	D20A M104T	1.666	1.1530	846.7
	H16E E61Q	2.343	0.8809	1258.3
	V91K D20A M104V	4.763	0.6466	430.0
	D20G	6.244	1.9392	825.3
	D20A H16R E61Q	20.720	7.0428	994.3
	D20W	20.924	16.1745	17.5
	N88K	23.315	14.1916	688.7

*The average EC50 values were calculated from non-linear regression analysis of IL-2 mutein dose response curves generated by pSTAT5 MFI readout. The average values with standard deviation (STDEV) were calculated from three donors. Calculated dMFI values at [IL-2] = 66.7 nM are also included*.

We defined class A as the group of muteins whose potencies (determined by pSTAT5 MFI EC50 values) are within 5-fold that of wildtype IL-2 and thus these muteins are only slightly attenuated. Class B muteins represent moderately attenuated muteins, where the EC50 values are between 5- and 25-fold higher compared to wildtype. Finally, class C muteins are highly attenuated, as their EC50 values are 25-fold or higher than that of the wildtype IL-2. The dose response curves of the muteins classified in this manner are shown in Figure 2B. As defined, both class B and class C muteins showed significant shift in EC50 values compared to wildtype IL-2, but class C muteins additionally demonstrated varying degrees of attenuation in maximal response (Rmax), as indicated by the plateau in dose response curves at high IL-2 concentration range. Attenuation of activity by EC50 and Rmax was not always linked, since there are muteins with >25-fold shift in EC50 but are still able to induce close-to-wildtype Rmax at high concentrations. Muteins such as D20G, D20A M104T, and D20A H16R E61Q represent this category. The weakest mutein (D20W), showed very little activity, indicated by its ability to induce activity in no more than 10% of the cells and barely detectable increase in pSTAT5 MFI. Thus, our mutein panel captures a wide spectrum of IL-2R signal, from wildtype level to almost complete lack of activity at a concentration as high as 200 nM *in vitro*.

### Attenuation of IL-2R Signaling Asymmetrically Impacts Treg and Non-Treg Cells

Interestingly, class A muteins, which showed very little attenuation in pSTAT5 response in Treg cells, demonstrated significant attenuation in CD25+ Foxp3– CD4 T cells (designated CD25+ Tconv in this paper for simplicity) as shown by the shift in MFI dose response curves (Figure 2B). Class B and C muteins showed even greater attenuation of activity in these cells. Although CD25 does not directly trigger intracellular signaling events, it can enhance the on-cell IL-2 activity by capturing IL-2 in solution and stabilizing the high affinity heterotrimeric receptor. Thus, we chose to compare the IL-2 activity on Foxp3+ (Treg) and Foxp3– CD4 T (Tconv) cells that are gated for positive CD25 expression. Nonetheless, CD25 levels on *ex vivo* Treg cells are significantly higher than those on unstimulated CD25+ Tconv cells (CD25 MFI, Supplementary Figure 1A) and as a result, a mutein's affinity to CD25 and/or its ability to aid in or hinder the assembly of the trimeric receptor may additionally impact its relative activity in Treg vs. non-Treg cells. To further narrow the differences in CD25 levels for this comparison, we also compared pSTAT5 response in CD25lo Treg cells gated to more similarly match the CD25 level on CD25+ Tconv cells (CD25+/lo Tconv in Supplementary Figure 3), but the differences in sensitivity of Treg and CD25+ Tconv cells persisted (Supplementary Figure 3). Additionally, to evaluate the signaling capacity of the muteins independently of their affinity for CD25, we evaluated the pSTAT5 response in cells that are negative for CD25. pSTAT5 responses in these cells, shown here by pSTAT5 data from CD25– Tconv cells (Figure 2B) and NK cells (Supplementary Figure 2), are significantly weaker compared to the CD25+ gated cells, and these represent IL-2 mutein activities generated solely through IL2RB and IL2RG We also note that the recombinant IL-2 showed stronger activity than the wildtype IL-2 in our molecular format, which may be due to aggregation (reported on the Proleukin label) and a resulting increase in avidity.

To further visualize how the attenuation impacted the muteins' activities in CD25+ Tconv cells compared to Treg cells, we plotted the percent pSTAT5+ and pSTAT5 MFI responses detected in CD25+ Tconv vs. Treg cells at two concentrations, at 66.6 nM and at 0.6 nM. The higher concentration corresponds to a concentration where most muteins reach their maximal response in Treg cells and therefore we consider it to represent a concentration of IL-2 that saturates the trimeric receptors on the cell surface. The lower concentration is 100-fold lower than this saturating concentration and for many muteins stands in the linear dose range. As indicated by the percent pSTAT5+ dose response data (Figure 2B), class A muteins are able to generate response in 100% of Tregs and close to 100% of CD25+ Tconv cells at saturation, while some of the class B and class C muteins are not able to induce response in 100% of cells even at the highest concentration tested, confirming that attenuation of STAT5 activation correlates with a significant loss in affinity to the receptor for these classes of muteins. Figure 2C demonstrates that the attenuation impacts the mutein activities in CD25+ Tconv vs. Treg cells non-linearly. For example, class A muteins induced maximal activity in both Treg and CD25+ Tconv cells at the high concentration, while the activity declined disproportionately at the lower concentration. At this non-saturating concentration, the muteins activity dropped dramatically in CD25+ Tconv while maintaining near maximal activity in Treg cells. Class B muteins displayed different behaviors at the high and the low concentrations. At saturation, these muteins showed significant attenuation selectively in CD25+ Tconv cells while they were still able to generate near maximal signal in Treg cells. At the lower concentration, their activity on Treg cells showed a range of attenuation while the activity in CD25+ Tconv cells remained maximally attenuated. Most of the class C muteins showed significantly attenuated activity in Treg and CD25+ Tconv cells at both concentrations. An exception was the H16E E61Q mutein, which contained a CD25 association mutation and showed a near-wildtype level of pSTAT5 response at the high concentration. These results show that the attenuation differentially impacts Treg vs. non-Treg cell subsets. IL-2-induced pSTAT5 response declined disproportionately faster in CD25+ Tconv cells with the attenuation, indicating that these cells exhibit heightened sensitivity to loss of IL-2 signal compared to Treg cells, a difference that was also observed for Treg and CD25+ Tconv cells gated for similar CD25 levels.

### Attenuation of Affinity to CD25 and IL2RB Synergistically Impacts Activity in CD25+ Cells

We measured the affinity of a subset of the muteins to CD25 and IL2RB to confirm our design rationale and to gain an insight into their activity and selectivity. As mentioned previously, most of the muteins contain a single or combination of mutations that are designed to disrupt the interaction with IL2RB. The muteins containing E61Q were designed to disrupt the interaction with CD25 to evaluate the additive impact on the IL-2 signal. Consistent with this design goal, the muteins that contain E61Q exhibit significant increase in their EC50 values (156.9–651.9 nM), suggesting weaker affinity to CD25. In contrast, muteins that exclude E61Q demonstrate similar affinity to CD25 as indicated by their EC50 values (6.0–43.1 nM, Table 2) obtained by SPR that was within the range observed for wildtype IL-2 (19.7 nM). Wildtype IL-2 binds to IL2RB only very weakly and in cells is far more likely to bind the IL2RB:IL2RG heterodimer rather than IL2RB alone. Nonetheless we evaluated binding activity of the muteins to IL2RB to confirm the attenuation. Due to the very weak signal measurable for IL-2:IL2RB interaction in this assay, we assessed binding of the muteins to IL-2RB relative to that of wildtype IL-2 (WTIL2-Fc) at a high fixed concentration (1,000 nM) (Table 2). Consistent with our design goal, E61Q by itself had very little impact on affinity to IL2RB, while all muteins containing IL2RB-attenuating mutations showed significant loss of binding. Among them, the class A muteins retained higher binding activities (V91G), while class B and some of the class C muteins retained trace amount of binding. The weaker class C muteins showed complete or almost complete loss of activity. These data confirm that, consistent with our design goal, IL-2 mutein activity correlated with its affinity to IL2RB. More importantly, these data show that Treg cells can induce relatively normal pSTAT5 response despite significant loss in binding to IL2RB, presumably due to a significant contribution of CD25 in the formation of the receptor complex that facilitates downstream signaling events and STAT5 activation.

**Table 2:** Relative binding affinity of IL-2 muteins to CD25 and IL2RB.

	**CD25**	**IL2RB**	
**Mutein**	**EC50, nM**	**% of WT binding**	**Class**
V91K D20A M104V	6.0	1.1	C
H16R	7.2	2.9	B
V91D	11.2	1.2	B
V91G	13.8	8.5	A
**WTIL2-Fc**	**19.7**	**101**	A
D20W	43.1	0.9	C
D20A H16R E61Q	156.9	−3.7	C
D20G	175.0	4	C
H16E E61Q	310.8	1	C
E61Q	651.9	98	A

*A subset of the mutein panel was evaluated for both CD25 and IL2RB relative binding activity using alternate assays. For CD25, EC50 values were determined from titrations of individual muteins against immobilized receptor by SPR binding assay as outlined in the methods. For IL2RB, relative binding affinity was approximated via the steady state binding RU level observed at a nominal mutein concentration of 1,000 nM where the values are reported as a percentage of the response observed with wildtype IL-2 (WTIL2-Fc) binding to immobilized IL2RB. WTIL2-Fc values are highlighted in bold as reference*.

**Figure 3:**
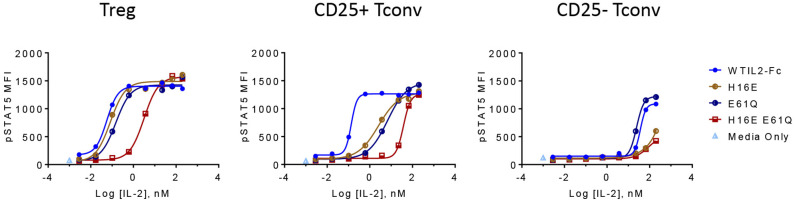
CD25 and IL2RB attenuation synergistically impacts IL-2 mutein activity and selectivity in CD25+ cells. The activity of IL-2 muteins containing a single mutation that slightly reduces affinity to CD25 (E61Q) or to IL2RB (H16E), or both (H16E E61Q), is shown as pSTAT5 MFI at increasing IL-2 mutein concentrations in CD25hi Treg cells, CD25+ Tconv cells, and CD25– Tconv cells. Shown are representative of three donors.

The CD25-attenuating E61Q mutation by itself had only a minor impact on overall activity as shown by the pSTAT5 EC50 and Rmax values in Treg cells (Figure 3), indicating that the levels of CD25 on Treg cells are high enough to tolerate partially reduced CD25 affinity in IL2-Fc homodimers. In CD25+ Tconv cells, with reduced CD25 levels, E61Q induced an attenuated pSTAT5 signal compared to wildtype IL-2, while in Tconv cells that were gated to exclude CD25-positive cells, E61Q retained activity similar to wildtype. This indicates that the attenuating effect of E61Q is dependent on and is sensitive to CD25 levels. In cells that express CD25 (Treg and CD25+ Tconv), E61Q exerted a significantly additive effect on attenuation when combined with an additional mutation that disrupts the interaction with IL2RB, since the potency of the mutein containing double mutations, H16E E61Q, is more than 30-fold decreased compared to that of the H16E single mutein. In contrast, in cells that lack CD25, H16E E61Q showed the same activity profile as did H16E single mutein. These results suggest that CD25– and IL2RB-directed mutations can additively attenuate IL-2 signal in CD25+ cells. D20W, which induced barely any detectable pSTAT5 signal, still exhibited normal affinity to CD25 suggesting that this mutein retained protein stability and integrity.

### Treg Cells Tolerate Significant Loss of IL-2 Activity in Generating Biological Responses

**Figure 4:**
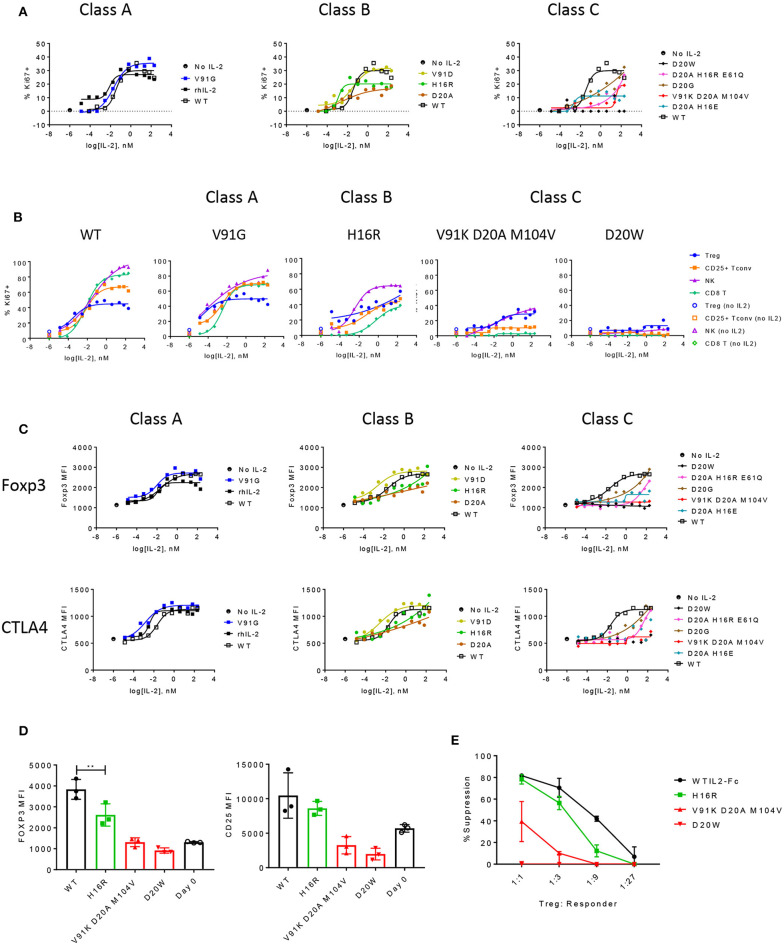
The functional responses of Treg cells are quantitatively sensitive to attenuated IL-2 signal. **(A)** Proliferation of Treg cells in response to select muteins from each class was measured in human PBMC assay and shown as percent Ki67-positive cells in CD25+ Foxp3+ CD4-gated Treg cells. Shown are data representative of four donors. **(B)** Proliferative responses measured as percent Ki67 positives of Treg, CD25+ Tconv, NK (CD56+ CD3–), and CD8 T cell gated subpopulations to increasing concentrations of wildtype or IL-2 muteins are compared in a total PBMC assay, for select muteins from each class. Shown are representative of data from four donors. **(C)** IL-2 mutein activity on induction of Foxp3 and CTLA4 in Treg cells are shown, represented as MFI of Foxp3 or CTLA4 in Foxp3-positive and CTLA4-positive gated Treg cell populations, respectively. **(D)** Treg phenotype before and after stimulation. Purified human Treg cells were stimulated with anti-CD3 and wildtype or IL-2 mutein at 66.7 nM for 3 days and analyzed for Foxp3 and CD25 expression. Day 0 unstimulated Treg cells analyzed at the same time to show baseline expression of these markers. Each color represents the mutein class or day 0: wildtype (black closed circles), class A (blue), class B (green), class C (red), and day 0 (black open circles). Shown are combined data from three different donors. ** represents *p*-value of 0.005 from a one-way ANOVA analysis. **(E)** Treg suppression assay. Purified human Treg cells that were pre-stimulated with anti-CD3 and IL-2 mutein were co-cultured with purified CD8 T cells from an unmatched donor at various ratios. CD8 T cells were stimulated with CD3+CD28 activation beads and Treg-mediated suppression was measured by activation marker induction on CD8 T cells on day 1. These values were converted to percent suppression, each point representing the average of values from two donors. Error bars indicate standard deviation.

Using *in vitro* assays, we evaluated how effectively these IL-2 muteins induced distal biological responses and attempted to define a threshold IL-2 signal that would selectively induce cell proliferation, upregulate activation markers, and enhance suppressive activity in Treg cells. We performed proliferation assay with human PBMC in the presence of titrating doses of IL-2 muteins and measured the expression of the proliferation marker Ki67. As shown in Figure 4A, class A mutein V91G induced similar Ki67 response as did wildtype IL-2 in Treg cells. Among the class B muteins, V91D, the most potent mutein in this class, behaved more like class A muteins and showed very similar activity as the wildtype control in all measurements from this assay (Figure 4C). For this reason, we re-classify V91D and N88D (Supplementary Figure 4) as class A muteins (shown as A/B in Table 1) for subsequent analyses. H16R, with a 20-fold reduction in potency, did show a significant loss in activity in inducing Treg cell proliferation. These results suggest that Treg cells retain full response to IL-2 with up to 6–20-fold loss in potency. The rest of class B and class C muteins showed varying degrees of reduced response across a wide concentration range compared to wildtype IL-2. The weakest mutein, D20W, did not induce any detectable Ki67 expression in Treg cells.

In this same assay, we also evaluated the IL-2-induced proliferative responses of additional cell subsets to determine whether CD25+ Tconv, CD8 T, and NK cells showed similar sensitivity to IL-2 attenuation as did Treg cells. Wildtype IL-2 and all class A muteins induced higher proliferative responses in CD25+ Tconv, CD8, and NK cells compared to Treg cells at IL-2 concentrations higher than ~10^−2^ nM (Figure 4B and data not shown), while inducing comparable or weaker response at the lower concentration range (<10^−2^ nM). Consistent with the observation that the class A muteins generated weaker pSTAT5 response in non-Treg cells compared to wildtype IL-2, the muteins induced diminished Ki67 response in CD8 and NK cells (Figure 4B). Interestingly, class A mutein effect on CD25+ Tconv proliferation was not diminished compared to wildtype IL-2. We think that this may be due to an autocrine effect of endogenously produced wildtype IL-2 on pre-stimulated CD4 T cells, which may mask signaling deficit of a mildly-attenuated class A mutein. Class B muteins showed significantly reduced response in CD25+ Tconv and CD8 T cells to the extent that Treg cell response was similar to or better than non-Treg T cell responses. Interestingly, NK cells still proliferated better than Treg cells, even though the IL-2 mediated pSTAT5 response in NK cells is significantly weaker compared to Treg and other cell types (Supplementary Figure 2). One possible explanation for such a disproportionately sensitive response of NK cells is that other cell types present in this assay indirectly aid in NK cell proliferation, by producing additional cytokine(s) or by capture and transpresentation of IL-2 via CD25. Class C muteins showed further attenuation across all cell types, but most significantly in CD25+ Tconv and CD8 T cells, thus these muteins preferentially induced Treg cell proliferation even at high concentrations. D20W did not induce significant response in any of the cell subsets, consistent with the minimal pSTAT5 signal it induces. These data together suggest that CD25+ Tconv and CD8 T cells require higher IL-2 activity than do Treg cells, since even a modest degree of attenuation significantly reduced IL-2 mediated proliferation in these cells while exerting little impact on Treg cells.

Previously published studies have shown that Treg cell lineage and phenotypic markers such as Foxp3 and Helios and T cell activation markers such as CTLA4, ICOS, and GITR, are expressed at high levels in Treg cells and correlate with stability of Treg cell phenotype and/or suppressive function [reviews by Rudensky (46) and Elkord (47)]. Importantly, Foxp3 expression is thought to be regulated directly by IL-2 (21, 48), thus this represents one mechanism by which IL-2 enhances Treg cell phenotype and function. Therefore, we evaluated the muteins' ability to increase the expression of Foxp3 and other markers associated with Treg cell phenotype and function. As shown in Figure 4C, wildtype IL-2 and class A muteins induced robust expression of Foxp3 and CTLA4 in an IL-2 dose-dependent manner, while class B and C muteins demonstrated varying degrees of attenuation. Some muteins in these classes were able to induce a nearly wildtype level of expression when pushed to high concentration range despite dramatic attenuation in potency indicated by EC50 values; others induced no significant response throughout the entire concentration range. Overall the muteins exhibited parallel activities toward Foxp3 and CTLA4 (and others, data not shown) induction. In this assay, where we could directly compare proliferation and phenotypic marker expression simultaneously, we observed that our weaker class C mutein (V91K D20A M104V) was unable to increase Foxp3 or CTLA4 expression at all, even though it was able to induce Treg cell proliferative response at the highest concentration. This suggests that the threshold IL-2 activity required to induce Treg cell proliferation may be lower compared to that for the Foxp3 or CTLA4 expression.

The ability of muteins to enhance Treg cell fitness to suppress immune response was assessed in an *in vitro* Treg suppression assay. In order to minimize the overpowering effects of wildtype IL-2 produced by activated T cells, we assessed early readout of T cell activation in CD8 T cells co-cultured with purified Treg cells that were first stimulated with IL-2 muteins. Due to the limited number of Treg cells that could be purified from each donor, we evaluated only a representative mutein from classes B and C and compared their effects against those induced by wildtype IL-2 or D20W mutein, which we used as a negative control. As shown in Figure 4D, Foxp3 and CD25 induction by IL-2 muteins in purified Treg cells correlated with the mutein activity as previously shown in the proliferation and pSTAT5 assays. Specifically, H16R, a class B mutein, induced attenuated expression of Foxp3 and CD25 compared to wildtype IL-2, while both class C muteins, V91K D20A M104V and D20W, resulted in no enhancement of expression of these key markers compared to day 0 when the Treg cells were isolated. Similar trends were observed for CTLA4 (Supplementary Figure 5A). We evaluated early activation response of allogeneic CD8 T cells in the presence of these pre-stimulated Treg cells and report that the muteins demonstrated variable degrees of attenuation compared to wildtype IL-2 in supporting Treg-dependent suppression (Figure 4E; Supplementary Figure 5B). Interestingly, H16R was nearly as effective as wildtype IL-2 in inducing full suppressive function despite the modest but significant attenuation it had shown in inducing Foxp3 and CTLA4 expression (Figure 4D; Supplementary Figure 5). Nonetheless, Treg cells stimulated in the presence of H16R showed weaker activity at lower Treg:CD8 T ratios indicating that the functional enhancement is sensitive to relative IL-2 activity. Showing a similar trend, V91K D20A M104V induced attenuated but significant level of suppression despite its inability to induce Foxp3, CD25, and CTLA4 expression in Treg cells.

**Figure 5:**
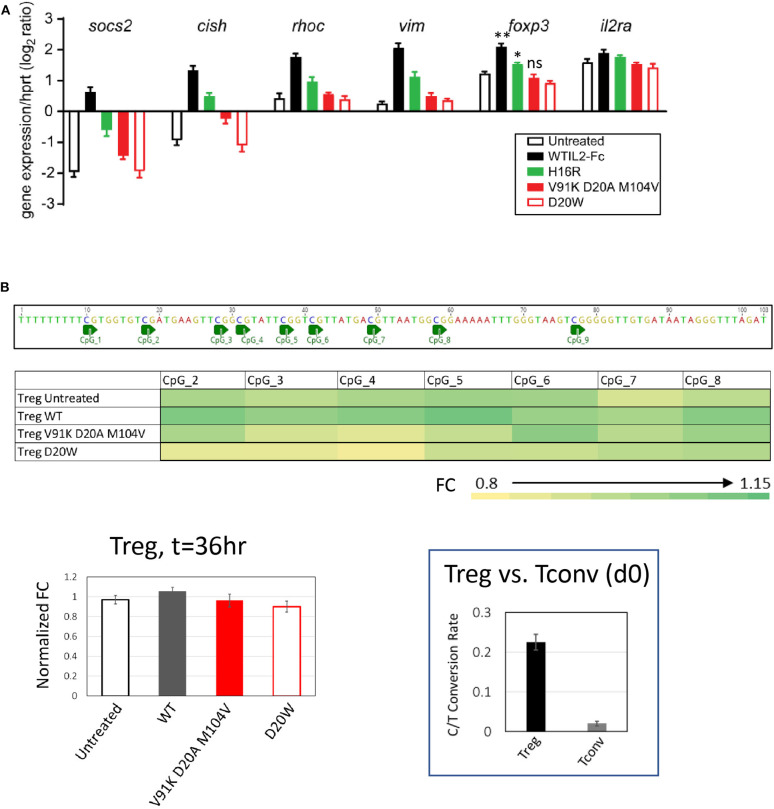
Treg-associated gene response is quantitatively sensitive to attenuated IL-2 mutein activity. **(A)** Transcript levels of *socs2, cish, rhoc, vim, foxp3*, and *il2ra* were quantified by Taqman assay in purified human Treg cells stimulated with various IL-2 muteins for 36 h. The results represent the mean ± SD of relative expression values (log2 ratio) for the indicated genes normalized to hypoxanthine-guanine phosphoribosyl transferase (*hprt*) in two independent donors with three replicates each. For *foxp3*, paired *t*-test was performed for each treatment group against the untreated sample and two-tailed *p*-values are indicated. **p = 0.0076; *p = 0.0165. **(B)** Methylation status of *foxp3* CNS2 region in human Treg cells cultured in IL-2 muteins. Methylation status was quantified as C-to-T conversion rate after bisulfite treatment. The fold change (FC) values were calculated based on the C-to-T conversion rates at individual CpG sites and normalized to Day 0 sample. The average FC values from three donors are plotted into heatmap. The average FC values calculated from the combined CpG sites were plotted as bar graph to show IL-2 mutein dependent effects. The BS4 amplicon sequence and CpG positions were illustrated above the heatmap. Shown in the inset is the representation of the overall C-to-T conversion rates for purified Treg and Tcon cells on day 0.

To further assess the impact of attenuated IL-2 activity on maintaining gene expression associated with Treg cell stability and function, we performed Taqman assays for a panel of genes that have been shown to be induced downstream of activated STAT5 in Treg cells (33), including suppressor of cytokine signaling 2 (*socs2)*, cytokine inducible SH2 containing protein (*cish*), Ras homolog family member c (*rhoc*), vimentin (*vim*), *foxp3*, and CD25 (*IL2ra)*. As shown in Figure 5A (and extended panel in Supplementary Figure 6), the transcript levels of multiple genes increased in response to wildtype IL-2, with attenuated muteins showing reduced response commensurate with their pSTAT5 signal. Importantly, we observed that the two class C muteins failed to induce significant accumulation of *foxp3* transcripts compared to wildtype IL-2 or the class B mutein, consistent with their lack of activity in maintaining Foxp3 protein expression in activated Treg cells (Figure 4D). This result indicates that transcript levels of Treg-associated genes correlate quantitatively with IL-2 signal.

Stable expression of Foxp3 is associated with demethylation of Treg-specific CpG methylation sites in the first intron of the *foxp3* gene, referred to as Treg-specific demethylated region (TSDR) (49, 50). Since we observed that *foxp3* transcript levels diminished with decreased IL-2 activity, we evaluated whether IL-2 activity is required for continuous maintenance of demethylation in TSDR by performing bisulfite sequencing analysis of genomic DNA from purified Treg cells stimulated with wildtype IL-2 of IL-2 muteins. As indicated by the calculated C/T conversion rate, TSDR is highly demethylated in purified Treg cells compared to Tconv cells across multiple CpG sites on day 0 (Figure 5B, inset). After 36-h stimulation with or without IL-2 muteins, TSDR remained fully demethylated regardless of the IL-2 activity, since the two weak class C muteins, as well as untreated sample, showed comparable activity as wildtype IL-2 in this assay. These results indicate that the maintenance of demethylation state of TSDR in purified human Treg cells is relatively resistant to attenuation or even loss of IL-2 activity, at least in an *in vitro* setting in this time frame.

### Induction of Treg Gene Signature Requires Stronger IL-2 Signal Than Do Other Treg Functional Responses

**Figure 6:**
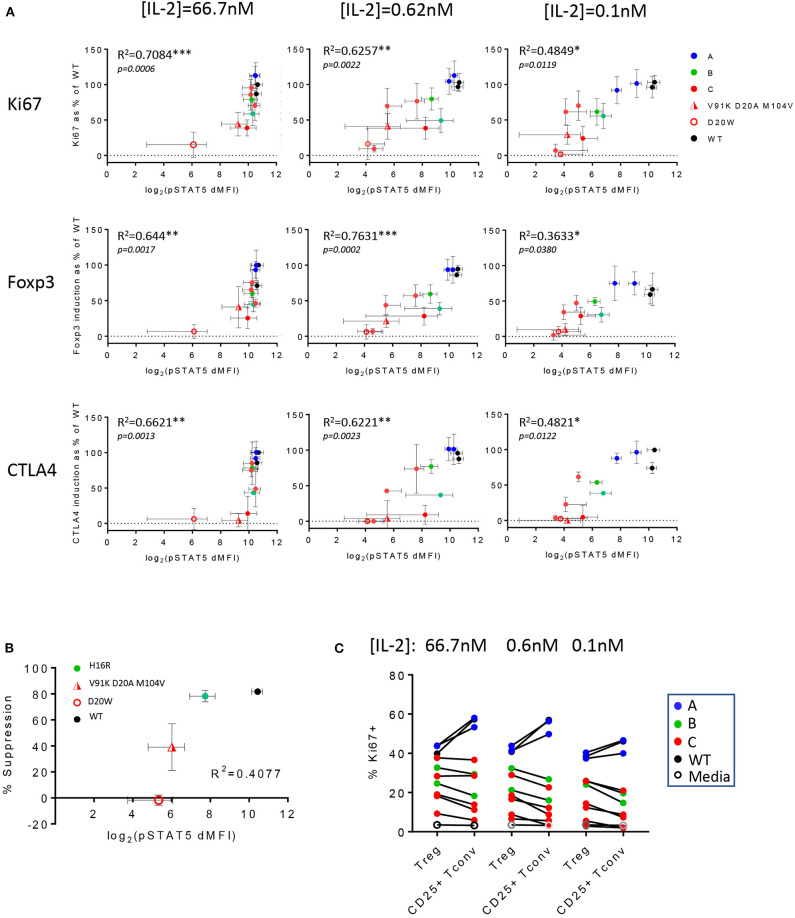
Treg cell functional responses are variably sensitive to IL-2 activity. **(A)** *In vitro* Treg cell proliferation (percent Ki67 positive of Treg cells) and Foxp3 and CTLA4 expression (MFI of Foxp3+ or CTLA4+ cells in Treg population) at IL-2 concentrations 66.7, 0.62, and 0.1 nM were converted to % response relative to wildtype IL-2 at 66.7 nM as the reference and plotted against pSTAT5 MFI (in log2 scale) values that were generated from the whole blood cell assay. The data are combined results from three different donors. The error bars indicate standard deviation. *R*^2^ values were calculated from Pearson *r* generated by correlation analysis, and the corresponding *p*-values are shown. Data points are color-coded by class, except the two weakest muteins (V91K D20A M104V and D20W). **(B)** Correlation plot of *in vitro* suppression and pSTAT5 signal in Treg cells. The y-axis represents the percent suppression of CD8 T cell activation read out by inhibition of activation marker expression in an 1-day Treg:CD8 T coculture assay at [IL-2] = 66.7 nM and Treg:CD8 T ratio of 1:1. One class B mutein (H16R) and the two weaker class C muteins (V91K D20A M104V and D20W) were evaluated against wildtype IL-2. The x-axis represents the pSTAT5 MFI data from the whole blood cell assay shown in log2 scale. The percent suppression represents the average ± SD values from two donors. The *R*^2^ value from the correlation analysis is shown. **(C)** Wildtype IL-2 and the muteins' activities on Treg vs. CD25+ Tconv are summarized in a pairwise comparison using proliferation as readout (indicated by % Ki67 positive) at three different IL-2 concentrations. The data points are color-coded according to the mutein class.

We generated correlation plots comparing the various measurements of biological responses against IL-2 mutein-induced pSTAT5 signal to interrogate whether a threshold IL-2 signal could be defined that leads to a detectable biological response. Since the biological assays were performed with pre-stimulated and rested PBMC, we evaluated pSTAT5 response of these cells with a subset of IL-2 muteins to establish that the muteins induced comparable pSTAT5 response on *ex vivo* cells (d0) as they did in stimulated and rested cells (d8) (Supplementary Figure 7A). The CD25 levels on Treg and CD25+ Tconv cells on day 8 were not vastly different from those on day 0 (Supplementary Figure 7B), thus we used the pSTAT5 readout on day 0 for the correlation analysis. As expected, we observed a positive correlation between the pSTAT5 signal and Treg cell proliferation, Foxp3 and CTLA4 expression throughout the range of different IL-2 mutein concentrations (Figure 6A). The IL-2 mutein activity also positively correlated with Treg cell survival, as indicated by the strong correlation seen between the total Treg cell frequency (% Treg of CD4 T) vs. pSTAT5 MFI and between Treg cell frequency vs. % Ki67+ Treg cells (Supplementary Figure 8). This indicates that the enhanced Treg cell proliferation and survival in response to IL-2 muteins leads to increased Treg cell number. The correlation was stronger at higher concentrations where the attenuation of the pSTAT5 signal would be expected to primarily reflect attenuation of the mutein activity at maximal possible receptor occupancy for the individual mutein. At lower concentrations where the mutations' impact on receptor binding and total occupancy may additionally factor in, the correlation was not as strong. In this analysis, class A muteins showed dramatic losses in pSTAT5 signal across the concentration range (66.7–0.1 nM), but it did not result in a corresponding reduction in biological responses. In fact, V91D was able to induce near maximal Ki67, Foxp3, and CTLA4 expression, despite over 67% reduction (estimated by the difference in pSTAT5 MFI) of pSTAT5 signal compared to wildtype IL-2 at 0.1 nM. These analyses also confirmed that class B and C mutein activities were reduced compared to class A muteins, as indicated by both pSTAT5 and biological responses, but similarly to class A muteins, there was a significant dissociation between the attenuation of activity represented by pSTAT5 readout vs. the effects on biological readout. Thus, although pSTAT5 MFI signal decreased significantly from 66.6 nM, 0.62 nM, to 0.1 nM, similar levels of the Ki67, Foxp3, and CTLA4 expression were maintained across all concentrations for individual muteins. This suggests that Treg cells are able to respond productively downstream of STAT5, as defined by their ability to proliferate and upregulate activation markers, to a wide range of IL-2 activity and that they can tolerate a significant degree of attenuation in IL-2 activity.

We took a closer look at these correlation plots, focusing on the weaker muteins for their ability to induce biological responses at lower concentrations. At 0.62 and 0.1 nM, the weakest muteins were not able to enhance Foxp3 and CTLA4 expression, despite generating small but significant pSTAT5 response over the baseline, establishing a threshold pSTAT5 signal that is required for induction of these markers (Figure 6A). Interestingly, some of these same muteins still induced significant Treg cell proliferation in this concentration range, suggesting that the threshold IL-2 activity for Treg cell proliferation is lower.

Similarly, attenuated muteins were able to support the suppressor function of Treg cells in an *in vitro* assay, but these activities were attenuated compared to wildtype IL-2 and ranked consistently with their pSTAT5 response (Figure 6B). In this assay, V91K D20A M104V demonstrated significant activity toward sustaining Treg cell suppressor function without a concomitant upregulation of Foxp3 (Figure 4D) and other activation markers (Supplementary Figure 5A), suggesting that IL-2 may be able to enhance Treg cell function independently of its effects on at least a subset of the canonical Treg cell markers. Finally, we observed that all of the class B and C muteins induced notably skewed Treg:CD25+ Tconv cell responses with preferential Treg cell response across the IL-2 concentration range, which distinguished them from the class A muteins which, like wildtype IL-2, induced greater Teff responses (Figure 6C). Thus, the Treg:CD25+ Tconv selectivity appeared to correlate with the degree of attenuation in activity, rather than the specific residues that are mutated.

**Figure 7:**
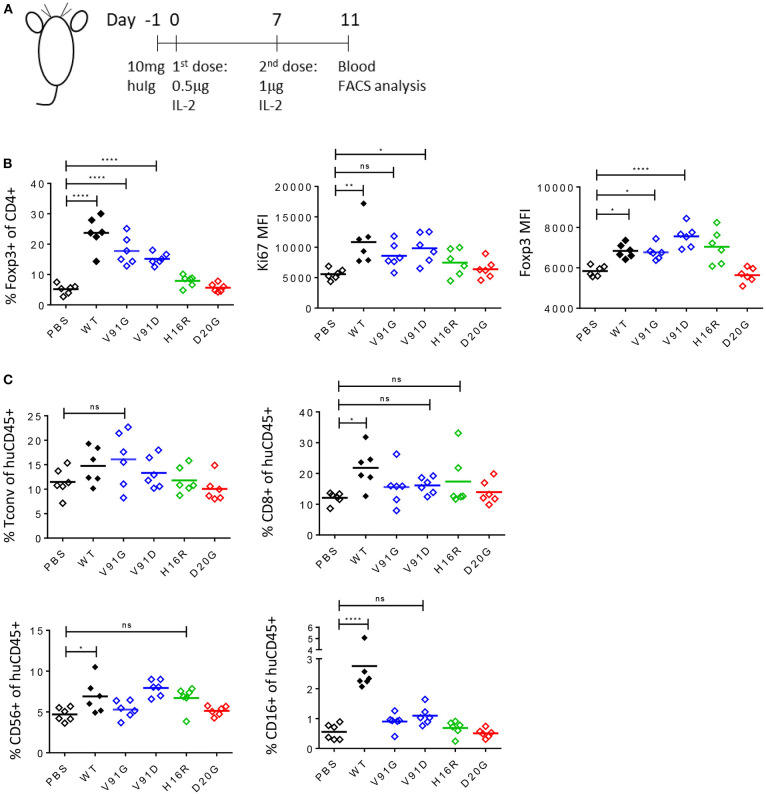
Attenuated IL-2 muteins induce Treg cell expansion with enhanced Treg:NK selectivity *in vivo*. **(A)** Study design schematic. One day before the first dose, humanized NSG mice were treated with 10 mg of human IgG. On day 0, wildtype IL-2 or IL-2 mutein was dosed s.c. at 0.5 μg per mouse, followed by a boost on day 7 at 1 μg per mouse. Blood was analyzed 4 days after the second dose, on day 11. Six animals per group were treated. Shown in the graphs, PBS-treated group served as vehicle control, and the muteins are color coded to represent class A (blue), B (green), and C (red). Ordinary one-way ANOVA analysis was performed to determine statistical significance of the comparisons between the wildtype- or IL-2 mutein-treated group and the PBS-treated group. **(B)** Attenuated IL-2 muteins induce human Treg cell response *in vivo*. Treg cells are gated based on Foxp3 expression and the representation of Treg cells are shown as percent of Foxp3+ in CD4 T cells. The levels of Ki67 and Foxp3 expression on Treg cells are shown as Ki67 MFI and Foxp3 MFI, respectively. *p < 0.02; **p < 0.0025; ****p < 0.0001. **(C)** The sizes of the various non-Treg cell compartments post IL-2 treatment are shown as percent of human CD45+ cells; Tconv (Foxp3– CD4 T), CD8 T, and CD56+ CD3– and CD16+ CD3– NK cell data are shown. *p < 0.04; ****p < 0.0001.

We further evaluated the ability of these muteins to induce Treg cell response *in vivo* using the humanized NSG (huNSG) mouse model, where human lymphocytes derived from the grafted human hematopoietic stem cells (huHSC) circulate in animals that lack their own lymphocytes. As shown in the study design schematic, we pre-treated huNSG mice with human gamma globulin to block non-specific binding of our molecules and dosed them twice with PBS, wildtype IL-2, or IL-2 mutein and analyzed the blood 4 days after the second dose (Figure 7A). We chose a low dose, at total of 1.5 μg, which we had determined with the wildtype molecule was a dose that induced a robust Treg cell expansion with minimal effect on Tconv and other non-Treg cells (data not shown), thus mimicking the effect of the low-dose IL-2 therapy in human patients. As shown in Figure 7B, wildtype IL-2 induced nearly a 5-fold increase in Treg cell number as indicated by the increased percentage of Foxp3+ CD4 T cells among total CD4 T cells, compared to the PBS-treated group. The two class A muteins showed a slightly reduced but still significant increase, while H16R, a class B mutein, showed a trend toward an increase but the effect was not statistically significant at this dose. D20G, a class C mutein, did not induce a response. These trends are conserved in the expression of Ki67 and Foxp3, as class A and B muteins showed a trend toward increased expression while the class C mutein showed responses that are comparable to the PBS-treated group. Importantly, wildtype IL-2, which robustly increased Treg cell number at this dose, showed a modest increase in Foxp3 expression. Thus, consistent with the *in vitro* data, Treg proliferative response appeared to be more sensitive to weak IL-2 signal than induction of Treg-associated gene expression *in vivo*. Furthermore, consistent with the general lack of effects of wildtype IL-2 at this low dose on Tconv cells, the attenuated muteins also did not show any effect on this population of cells (Figure 7C). Importantly, all muteins tested in this study also demonstrated reduced activity toward CD8 and CD16+ NK cells. These data further confirm our *in vitro* results that the Treg-to-non Treg selectivity is enhanced with attenuated IL-2.

## Discussion

Treg cells drive dominant tolerance by inhibiting inflammatory responses mediated by multiple types of activated cells. Lack of, or reduced number of Treg cells, or their functional deficit, leads to aggressive lymphoproliferative autoimmune disease in human and mice, as exemplified by IPEX syndrome and *scurfy* phenotype, respectively. Conversely, increasing the number of Treg cells by adoptive transfer (19) or treatment with low-dose [review by Klatzmann and Abbas (51)] or attenuated IL-2 (13–16) reduces inflammation and improves disease. However, increasing the number of Treg cells may not be sufficient, based on the examples where the presence of (or even elevated numbers of) Treg cells does not result in reduced inflammation (52, 53). Without being mutually exclusive, several explanations for this gap may be considered. One is that wildtype IL-2 induces a much more potent response in inflammatory non-Treg cells such that it undermines any enhancement of Treg cells, even at low doses where the Treg:CD25+ Tconv selectivity is increased. Second possibility is that the IL-2-mediated effects on Treg cells are limited in active disease due to the presence of pro-inflammatory cytokines that have been shown to destabilize Treg cell phenotype and function (54–56) or because a gap in antigen specificity of IL-2-stimulated Treg cells confounds the targeting of these cells to appropriate tissue(s). Another explanation may be that the amount of IL-2 signal required to enhance the functional fitness of Treg cells is higher than that for inducing proliferation and/or to protect from apoptosis. In this scenario, an attenuated IL-2 signal such as that generated by low-dose IL-2 treatment may increase the number of circulating Treg cells with enhanced expression of the various Treg cell phenotypic markers, but it may not be sufficient to enhance their suppressor function. In all these scenarios, the ultimate effects of IL-2 stimulation is likely additionally influenced by synergistic contribution of signals mediated by T cell receptor, TGFβ and/or members of the TNF receptor family.

Our panel of engineered IL-2 muteins induced a wide range of pSTAT5 signal through the IL-2 receptor. Analysis of these muteins whose maximal activity is attenuated compared to wildtype IL-2 allowed us to assess the impact of attenuated signal beyond the limits of the narrow concentration range that normally induces Treg-selective effects. Our data confirm the basic rationale for why the previously reported examples of attenuated IL-2 demonstrate increased selectivity for Treg cells vs. CD25+ Tconv and CD8 T cells. Importantly, our data directly demonstrate that Treg, Tconv, and CD8 T cells possess intrinsically different sensitivity to IL-2 signal. We restricted our comparison to CD25+ gated populations to compare Treg vs. CD25+ Tconv cell responses, however, discrepancies in the relative levels of CD25 expression is likely to be a factor in the observed difference in sensitivity. In this scenario, our observation that class A muteins selectively retain maximal STAT5 response in Treg cells while demonstrating a significant attenuation toward CD25+ Tconv cells at saturated receptor occupancy underscores the contribution of CD25 to overall IL-2 activity in Treg cells, consistent with its known role in facilitating the capture of IL-2 and stabilizing the heterotrimeric receptor. For these muteins, elevated CD25 expression may be sufficient to compensate for the slight loss of affinity to the IL-2RB chain which limits the maximal signal. In this context, it is of relevance that CD25 expression on Treg cells has been reported to be reduced in some diseases (e.g., SLE), which may limit their sensitivity to far-attenuated IL-2 muteins. We also note that despite the attenuated pSTAT5 response, non-Treg cells proliferated disproportionately better than Treg cells in response to wildtype IL-2 and class A muteins *in vitro*. Thus, attenuating the downstream biological responses in these cell types requires much greater attenuation that is indicated by the pSTAT5 readout.

Our analysis showed a strong positive correlation between pSTAT5 signal and multiple downstream biological responses relevant for Treg cell expansion and function. This was not surprising given that much of Treg cell response has been shown to be driven by activated STAT5 (21). Interestingly, the correlation became weaker at lower concentrations, as the receptor occupancy fell below maximal level, and we made two observations. First, Treg cells tolerate a great degree of attenuation of IL-2 signal, indicating that the threshold IL-2 signal required to induced Treg cell response is much lower than previously expected. Second, although the muteins induced Treg cell response that was commensurate with their relative maximal pSTAT5 response, the absolute response did not change significantly over a wide concentration range. It should be noted that in these *in vitro* assays, IL-2 dependent enhancement of Treg cell proliferation requires additional signals, e.g., pre-stimulation via the antigen receptor. Thus, the threshold IL-2 signal indicated in this context is somewhat confined by its requirement for synergistic signals or priming of Treg cells that permit the downstream response. Nonetheless, this is likely relevant for Treg cell responses *in vivo*. Homeostatic maintenance and function of Treg cells require both TCR signal (57, 58) and IL-2 signal (33, 34) and these two signals likely synergize to enhance Treg cell function. Since Treg cells continuously interact with antigen presenting cells *in vivo* that provide TCR and other types of signals (57–61), our results directly show that the availability of IL-2 remains a key factor that controls the Treg cell response *in vivo*, even when it acts in synergy with additional pathways. Using an *in vitro* assay, we demonstrated that the attenuated IL-2 muteins enhanced Treg cell suppressor activity that also correlated with their biochemical activity quantified by the pSTAT5 signal. Surprisingly, V91K D20A M104V, a mutein with a >25-fold reduction in potency, was still able to significantly enhance Treg cell fitness to inhibit activation of effector T cells, even though this same mutein showed hardly any activity in enhancing the expression of Treg cell markers such as Foxp3, CD25, and CTLA4. These results together suggest that, somewhat contrary to our initial hypothesis, a stronger IL-2 activity is required to maintain the high level expression of lineage and activation markers associated with Treg cell stability than to enhance Treg cell proliferation and suppressor function. In human Treg cells, it has been shown that Foxp3 expression is required but not sufficient to maintain Treg cell phenotypic stability and suppressor function [review by Bacchetta et al. (62)]. IL-2 provides a key signal, via STAT5 activation, to induce and maintain Foxp3 transcription. However, IL-2 is likely to influence additional pathways in Treg cells that are distinct from its effects on Foxp3 expression, as has been suggested previously (63) and in this context is interesting to consider that IL-2 may be able to support pathways required for Treg cell function independently of its effect on the Foxp3 expression. Since our study focused on evaluating the IL-2 mutein activity in mature human Treg cells purified from normal healthy people, our data speaks to the relatively stable functional phenotype of mature Treg cells that persists in response to attenuated IL-2 signal, even as the *foxp3* transcript level declines. Additional studies are required to address whether *de novo*/*in vivo* activity of IL-2 muteins on Foxp3 expression vs. suppressor activity shows similar disconnect. We also observed that the *foxp3* enhancer region remained highly demethylated in Treg cells treated with weak muteins, even though the *foxp3* transcript and protein levels showed significant reduction in these cells. These results suggest that, in highly purified Treg cells from normal healthy people, IL-2 signal plays a more prominent role in stabilizing *foxp3* transcript than in regulating the methylation status of these enhancer region(s).

From a therapeutic view point, it is difficult to predict the Treg cell number or the level of suppressor function that would sufficiently inhibit pathogenic inflammatory response *in vivo*. However, if the threshold requirements for IL-2 in enhancing or maintaining the Treg cell number, function, and lineage stability are indeed variable, the *in vivo* efficacy of an attenuated IL-2 mutein or low dose IL-2 in disease is more likely to be limited by its inability to maintain the stability of Treg cells, as may be indicated by the expression of Foxp3 and other markers, and by its Treg:CD25+ Tconv selectivity, rather than insufficient activity toward immediately enhancing Treg cells' suppressor function. Addressing this question would require evaluation of these muteins' activities in *in vivo* disease models, which we were not able to perform due to anti-drug antibody (ADA) response to human IL-2 muteins containing human Fc in mice and the difference in the relative potencies of human IL-2 muteins on mouse Treg and CD25+ Tconv cells (data not shown).

IL-2 is required for Treg cell homeostasis and function, and it also acts as a potent driver of Teff and NK cell responses. Wildtype IL-2 is extremely efficient in assembling and productively engaging its receptor, and it exhibits a narrow linear activity range. As a result, the means to control the level of IL-2 signal with wildtype IL-2 is limited. Our study showed that, in designing the next generation of engineered IL-2 as a therapeutic, attenuated IL-2 as a class is superior to wildtype IL-2 in enhancing the Treg:non-Treg selectivity and that a robust activity in increasing Treg cell function and stability are key factors that should be considered in addition to expansion in number. With appropriately attenuated muteins, we anticipate being able to generate a much more controlled Treg-selective IL-2 responses over a considerably wider dose range than with wildtype IL-2 in patients. Additionally, these IL-2 muteins will serve as powerful tools to explore the biology of IL-2 in the many facets of its contribution to immune regulation.

## Materials and Methods

### Human IL-2 Mutein Design and Material Production

Structure based designs were evaluated through analysis of the 2B5I with IL-2 cytokine receptor complex (CD25, IL2RB, IL2RG, and IL-2) using Pymol (The PyMOL Molecular Graphics System, Version 2.0 Schrödinger, LLC). Nucleotide changes were introduced using the Geneious software suite. gblock fragments from IDT-DNA were cloned into a mammalian expression vector using Golden Gate cloning system (ThermoFisher). Constructs encoding effector Functionless Fc fusions (64) of IL-2 variants were stably integrated into CHO K1 cells and expressed at 32°C in a 6-day batch production. The proteins were purified from clarified culture media using MabSelect SuRe affinity column (GE Healthcare) followed by HiTrap Desalting (GE Healthcare), and final purification was performed on a Superdex 200 Increase size exclusion column (GE Healthcare). Proteins were stored at 0.5–3 mg/ml in 10 nM Acetate pH 5.2, 100 M NaCl for biophysical and functional assays.

### Human Whole Blood Phospho STAT5 Assay

*In vitro* STAT5-phosphorylation analysis was performed using human whole blood collected from healthy volunteer with informed consent under Amgen Research Blood Donor program. Following incubation with IL-2 muteins for 30 min at 37°C, samples were treated with pre-warmed Lyse/fix buffer (BD Biosciences) and incubated for further 10 min at 37°C. Fixed and lysed blood sample were subsequently stained for CD25 (2A3, BB515, BD Biosciences), and permeabilized by incubating overnight at −20°C in pre-chilled Perm Buffer III (BD Biosciences). The next day, samples were stained for CD3 (UCHT1, Pe-Cy7, Invitrogen), CD4 (SK3, BUV395, BD Biosciences), CD8 (SK1, BV711, BioLegend), Foxp3 (259D, AF647, BioLegend), and pSTAT5 (47, PE, BD Biosciences). For NK cell panel, fixed and lysed blood samples were subsequently stained using CD25 (2A3, BV421, BD Biosciences), CD56 (HCD56, AF488, BioLegned), and CD16 (3G8, BV510, BioLegend) and then permeabilized by incubating overnight at −20°C in pre-chilled Perm Buffer III (BD Biosciences). The next day, samples were stained for CD3 (UCHT1, Pe-Cy7, Invitrogen), CD4 (BUV395, SK3, BD Biosciences), CD8 (BV711, SK1, BioLegend), Foxp3 (259D, PE, BioLegend), and pSTAT5 (47, AF647, BD Biosciences); data were acquired using BD FACSymphony (BD Biosciences) and analyzed using FlowJo software (v10.6.1, BD).

### Biacore Affinity Measurements

For analysis of binding to CD25, a standard SPR binding assay was employed. A CM5 (GE Healthcare) Biacore T100 chip was pre-conditioned according to the manufacturer's suggested protocol. Recombinant CD25 protein (R&D Systems, cat #223-2A/CF) was reconstituted as a 0.1 mg/mL solution in PBS, prior to dilution into 10 mM sodium acetate (pH = 4.5) immobilization buffer (GE Healthcare), for a final CD25 concentration of 1 μg/mL. Approximately 90 RU of ligand was coupled to the sensor surface using standard EDC/NHS coupling protocols provided by the manufacturer. For creation of a reference flow cell, activation and quenching of the sensor surface without ligand attachment was performed. For assay of the analyte panel, all proteins were prepared as 3-fold dilution series from 500 to 6.2 nM in running buffer (PBS + 0.05% Tween-20). Prior to data collection, regeneration scouting was performed, and surface stability of CD25 assessed after repeat injections of 50 nM WT IL2-Fc parent standard. CD25 surface activity post-coupling was estimated to ~50%. For binding assay, injection times of 60 s (500–55 nM) or 120 s (18–6.2 nM) were used to achieve steady state binding levels, followed by a 5 min dissociation phase prior to regeneration of the surface using 2 × 15 s pulses of 10 mM glycine^*^HCL (pH = 1.5). For each concentration series, duplicate injections were performed in sequence. A flow rate of 75 μL/min was used throughout the analysis. Data analysis was performed by double referencing each sensorgram against both reference flow cell and blank injections. Steady state binding (RU) levels were used during a global fitting routine of the entire dataset to a 4-parameter logistic function to derive EC50 values.

For analysis of binding to IL2RB, Biacore Series S, SA chip was pre-conditioned per the manufacturers suggestion prior to immobilization of minimally biotinylated recombinant human IL2RB-huFc (Sino Biological 10696-HO2H) to a density of between 400 and 500 RU. One untreated flow cell was utilized at a reference channel. For screening of the mutein analytes, all were prepared as 1,000 nM solutions in running buffer (PBS-P+, +0.2% BSA, +0.01% sodium azide). Analysis was conducted by injecting analyte for 40 s at flow rate of 30 μL/min, followed by 60 s dissociation. Regeneration of the active surface was achieved by 15 s pulses of 1 M MgCl_2_ in sodium Acetate (pH 5.5). Surfaces were tested as stable and binding deemed reproducible by repeated injection of the WTIL2-Fc protein control prior to initiation of the screen. Data were double referenced, and the maximum signal at steady state was recorded. The data were reported as % binding to the control, calculated as follows:

$$\begin{array}{l} \%\text{ control}=\left(\text{mean RU Sample}\right)/\left(\text{mean RU WTIL}2–\text{Fc control}\right)\\ \;\times 100 \end{array}$$

The mean of each sample was from duplicate injections; the controls were injected at least 4 times per run. RU was taken at binding maximum, or steady state with 1,000 nM concentration.

### Human PBMC Proliferation Assay

Total PBMCs were stimulated with α-CD3 antibody (OKT3, Biolegend) at 0.1 μg/ml for 2 days in complete RPMI 1640 media containing 2% heat-inactivated human AB serum (Sigma-Aldrich), washed, and rested in complete media for 5 days. On day 7, cells were harvested and 200 k cells were cultured in complete media containing only IL-2 mutein at titrating concentrations in a 96-well flat bottom plate. Five days later, cells were harvested and labeled with a viability dye (LIVE/DEAD Fixable Near-IR Dead Cell stain Kit, ThermoFisher Scientific) per manufacturer's protocol and stained for cell surface markers: CD3 (BUV805, BD Biosciences), CD4 (BUV395, BD Biosciences), CD8 (BV510, Biolegend), CD25 (BB515, BD Biosciences), and CTLA4 (a-CD152-PE/Cy7, Biolegend). Stained cells were fixed and permeabilized using the Foxp3 fix/perm buffer kit (eBioscience) and stained for Foxp3 (Alexa Fluor 647, Biolegend) and Ki67 (BV421, BD Biosciences). Samples were acquired on BD FACSymphony (BD Biosciences) and analyzed using FlowJo software.

The raw values of the percent Ki67-positive or Foxp3 and CTLA4 median fluorescence intensity were converted to percent response (% response) based on the values obtained from cells stimulated with wildtype IL-2 at 66.7 nM or media only (baseline). The equation that was used for conversion is as follows:

$$\begin{array}{l} \%\text{response}=(\text{experimental}-\text{baseline})/(\text{value at wildtype IL}\\ \;-2\;@\;66.7\text{nM}-\text{baseline})\times 100 \end{array}$$

### Human Treg Cell Purification and Stimulation

PBMCs isolated from healthy human donor leukopaks (AllCells) by density gradient centrifugation (Ficoll Paque Premium, GE-Healthcare) were used for isolating CD4+ CD127Low/- CD25+ Treg cells. CD4+ CD127Low/- CD25+ Treg cells were isolated from PBMC using EasySep human CD4+ CD127Low CD25+ Regulatory T cell isolation kit (STEMCELL Technologies). Isolated Tregs were cultured in tissue culture plate pre-coated with anti-CD3 at 2 μg/ml (OKT3, BioLegend) plus WTIL2-Fc, H16R, V91K D20A M104V, or D20W IL-2 muteins at 66.6 nM in RPMI1640 media containing 10% Heat-inactivated Human AB serum (Sigma) and supplemented with GlutaMAX, 1mM Sodium Pyruvate, Non-essential Amino Acids, 10 mM HEPES, 100 U/ml Penicillin and Streptomycin, β-Mercaptoethanol (all from GIBCO, Life Technologies) for 3 days at 37°C.

### *In vitro* Treg Suppression Assay

To evaluate the activity of IL-2-treated Treg cells, purified CD8+ T cells were used as responder cells in a Treg:CD8 T coculture system. CD8+ T cells were purified from PBMCs isolated from healthy human donor derived leukopaks (AllCells) using EasySep CD8+ T cell Negative selection kit (STEMCELL Technologies) and were cultured with purified and pre-stimulated (as described in the human Treg cell purification and stimulation section) human Treg cells at varying ratios, in the presence of anti-CD3/anti-CD28 magnetic DynaBeads (Invitrogen) added at 1:12 bead:T cell ratio. Suppression was measured by inhibition of activation marker CD25 expression on responder CD8+ T cells after overnight incubation at 37°C.

Percent suppression was calculated using changes in CD25 MFI as a measure of activation in responder CD8 T cells using the following formula:

$$\begin{array}{l} \%\text{Suppression}=100-[(\text{MFI in the presence of Treg}-\text{MFI }\\ \text{ in non-activated Responder only})/\\ \;(\text{MFI in activated Responder only}-\text{MFI in }\\ \text{ non-activated Responder only})\times \;100]. \end{array}$$

Statistical data analysis was performed using GraphPad Prism software (v7.04, GraphPad Software, Inc.). To compare the effect of different IL-2 muteins and WTIL2-Fc in Treg suppression assay, 2-way ANOVA test was used with Tukey *post-hoc* test.

### Taqman Analysis of STAT5-Inducible Genes

Total RNA was extracted from Treg cells stimulated under indicated conditions using Trizol reagent (Invitrogen) according to the manufacturer's protocol. cDNA was synthesized using Superscript III Reverse Transcriptase (Invitrogen). Quantitative PCR was performed using FAM-MGB labeled Taqman probes purchased from Invitrogen.

### Foxp3 Bisulfite Amplicon-Seq DNA Library Preparation and MiSeq Analysis of IL-2 Mutein Treated Treg Cells

CD4+ CD127low/- CD25+ Tregs were isolated from PBMCs from male donors using EasySep CD4+ CD127low/- CD25+ Regulatory T cell isolation kit as previously mentioned. Enriched Tregs were cultured in complete RPMI media containing 10% heat-inactivated human AB serum and WTIL2-Fc, H16R, V91K D20A M104V, or D20W at 66.6 nM for 36 h at 37°C. CD25– Tconv cells were also isolated from the unbound fraction of the Treg purification process and further enriched by CD25 bead depletion (Miltenyi) and cultured without any stimulation as a negative control.

Genomic DNAs were extracted using Gentra Puregene Cell Kit (Qiagen) protocol. Fifty nanogram gDNA was used for the bisulfite conversion following the protocol of EpiTect Bisulfite Kit (Qiagen). Five nanogram converted gDNA was amplified by PCR, and amplicons were purified using SPRIselect beads (Beckman Coulter) for next generation sequencing.

To prepare the Amplicon-Seq DNA library, we processed ~50 ng PCR products of each Bisulfite treated sample using the Nextera XT (Illumina: FC-131-1096) kit per manufacturer's suggested protocols for DNA library preparation. All samples were multiplexed using Illumina-supplied Index 1 and Index 2 primers followed by a 12-cycle PCR reaction. The DNA libraries were then cleaned up using the AMPure XP beads. Agilent TapeStation was used to perform quality control (QC) on PCR amplified libraries. All libraries were sized between 200 and 550 bp. Four nanometer amplified library of each sample was then pooled together from the DNA library and sequenced using the 300-cycle (MiSeq Reagent Kit v2) sequencing kit format. The paired-end sequencing was performed on the Illumina MiSeq and the results were analyzed on ArrayStudio.

### *In vivo* Activity Assay

Female humanized NSG (NOD scid gamma) mice (NSG mice reconstituted with human CD34 stem cells, JAX Laboratory) were randomized based on the percentage of human CD45 cell engraftment. Ten milligrams of human gamma globulin were injected into each mouse subcutaneously 1 day before (d −1) IL-2 mutein dosing to block non-specific binding to unoccupied human FcRs. Mice were dosed subcutaneously with 0.5 μg of various IL-2 muteins or control on day 0 and a second boost dose was given at 1.0 μg per mouse on day 7. Our choice for weekly dosing and analysis on day 4 post the boost was based on the prolonged PK/PD effects reported for a similar molecule in non-human primate (NHP) and nod.scid mice (65). Non-terminal retro-orbital bleeding was performed and 100 μL of whole blood was obtained from each mouse on day 11 (day 4 post boost) for FACS staining. Whole blood was stained with cell surface markers: CD3 (FITC, BD Biosciences), CD56 (PerCP, BD Biosciences), CD16 (PE-Cy7, BD Biosciences), CD25 (APC, Miltenyi), CD45 (APC-Cy, BD Biosciences), CD4 (V500, BD Biosciences) for 20 min before red blood cells were lysed in BD FACS lysing solution (BD Biosciences). Cells were then fixed and permeabilized, and stained for Foxp3 (PE, Biolegend) and Ki-67 (V450, BD Biosciences) using Foxp3 staining buffer set (eBioscience). Data were acquired on LSRII (BD Biosciences) and analyzed by Flowjo. For statistical analysis, ordinary one-way ANOVA analysis was performed using GraphPad Prism software (v7.04, GraphPad Software, Inc.).

All experimental studies were conducted under protocols approved by the Institutional Animal Care and Use Committee of Amgen. Animals were housed at Association for Assessment and Accreditation of Laboratory Animal Care International-accredited facilities (at Amgen) in ventilated micro-isolator housing on corncob bedding. Animals had access *ad libitum* to sterile pelleted food and reverse osmosis purified water and were maintained on a 12:12 h light:dark cycle with access to environmental enrichment opportunities.

## Data Availability Statement

All datasets generated for this study are included in the article/Supplementary Material.

## Ethics Statement

The animal study was reviewed and approved by Institutional Animal Care and Use Committee at Amgen.

## Author Contributions

AG performed majority of the experiments. DB designed and produced IL-2 muteins. KC performed the CD25 and IL2RB affinity measurements. M-ZW, JL, and C-ML performed the TSDR assay. Y-LH performed the *in vivo* studies with the humanized NSG mice. AC performed the Taqman analysis. SS conceptualized, performed experiments, and wrote the manuscript. All authors wrote the methods and figure legends for the data they generated.

## Conflict of Interest

All authors are current employees of Amgen and may own Amgen stocks. The authors declare that this study received support from Amgen, Inc. The funder had the following involvement with the study: study design.
