# Supplementary figures and images for: Defining the Threshold IL-2 Signal Required for Induction of Selective Treg Cell Responses Using Engineered IL-2 Muteins
Source: Front Immunol. 2020 Jun 5;11:1106. doi: 10.3389/fimmu.2020.01106 (PMC7291599; doi:10.3389/fimmu.2020.01106)

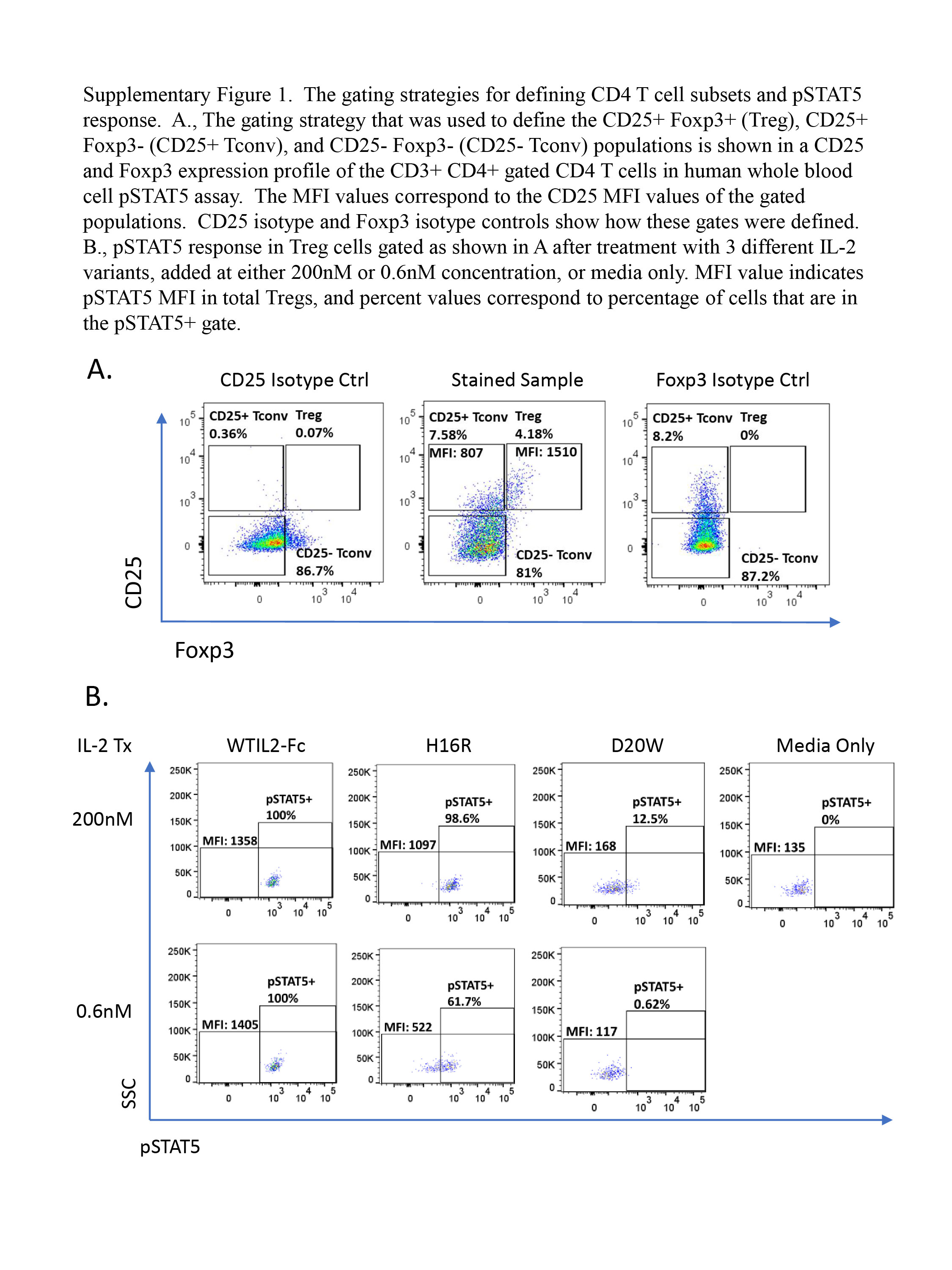

Supplement: Supplementary file 1 [file Image_1.jpg]

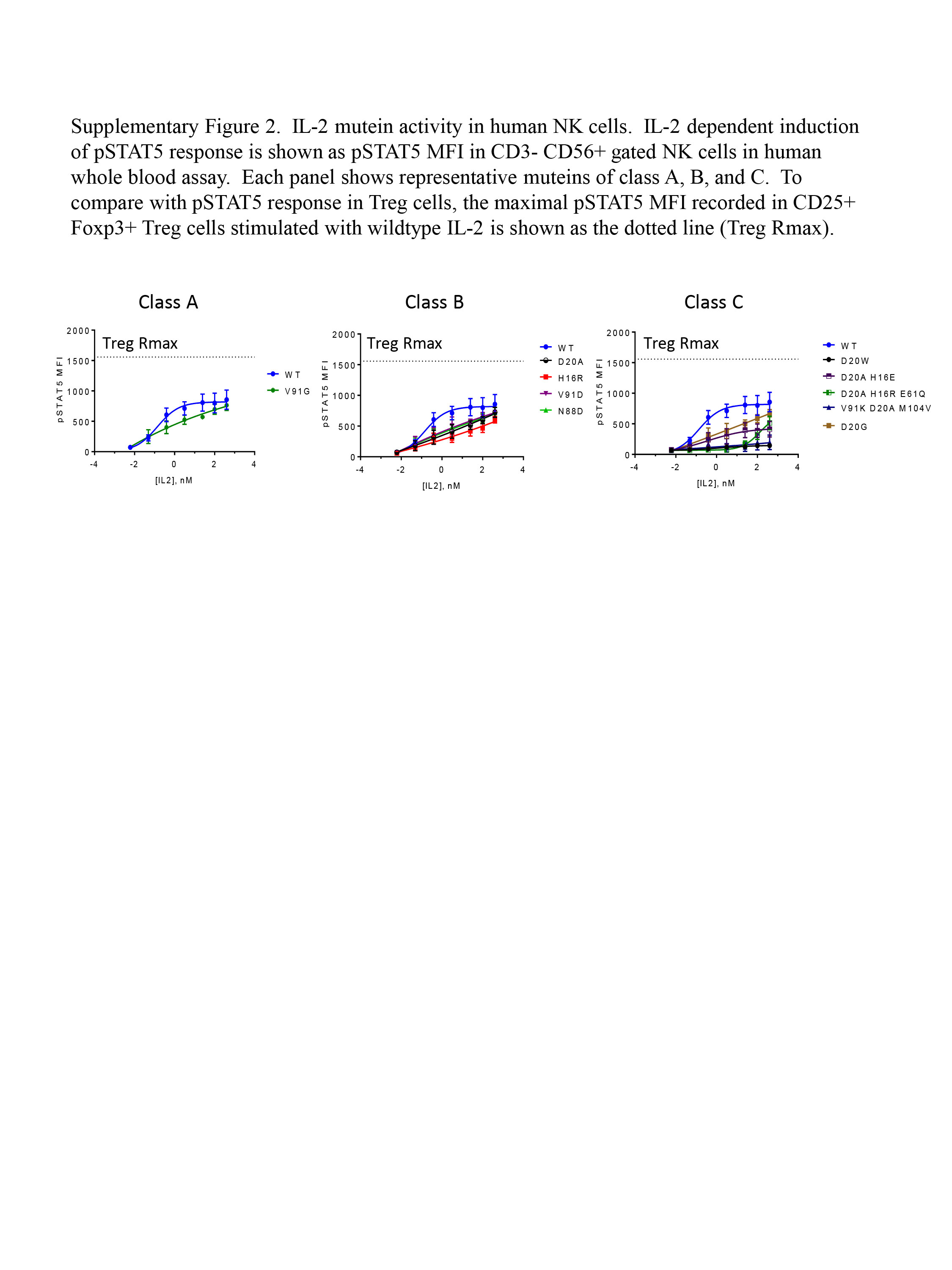

Supplement: Supplementary file 2 [file Image_2.JPEG]

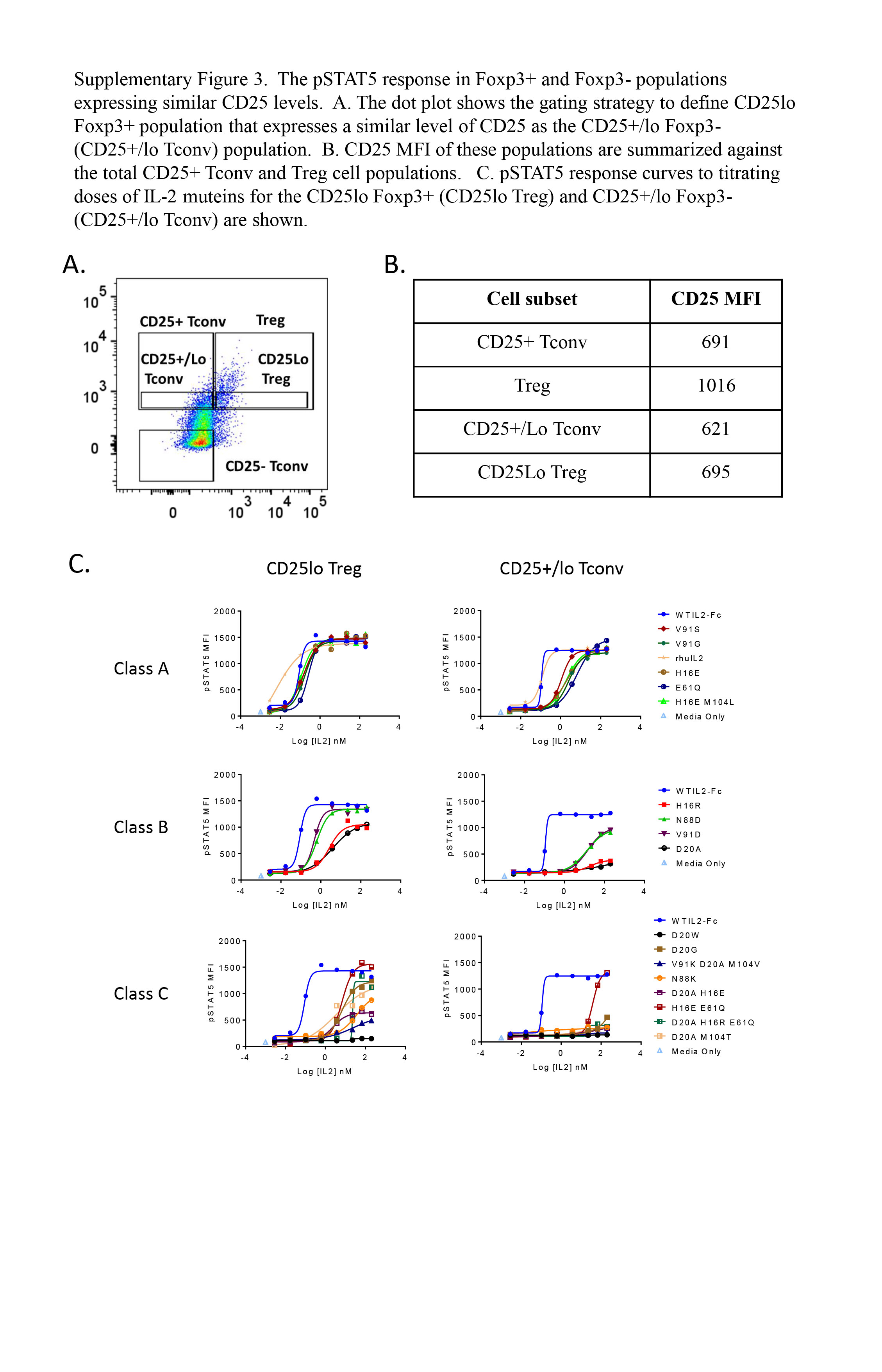

Supplement: Supplementary file 3 [file Image_3.JPEG]

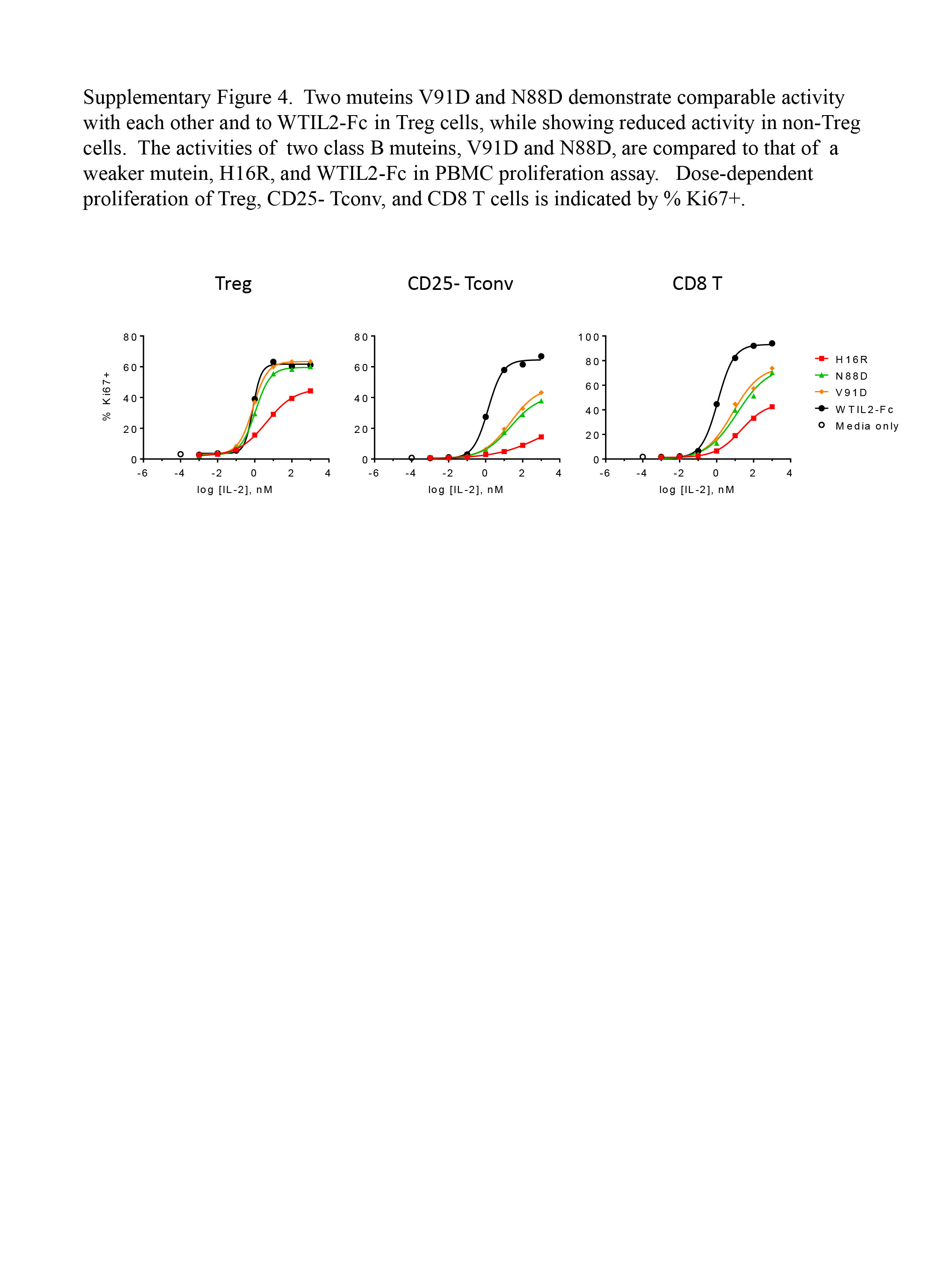

Supplement: Supplementary file 4 [file Image_4.JPEG]

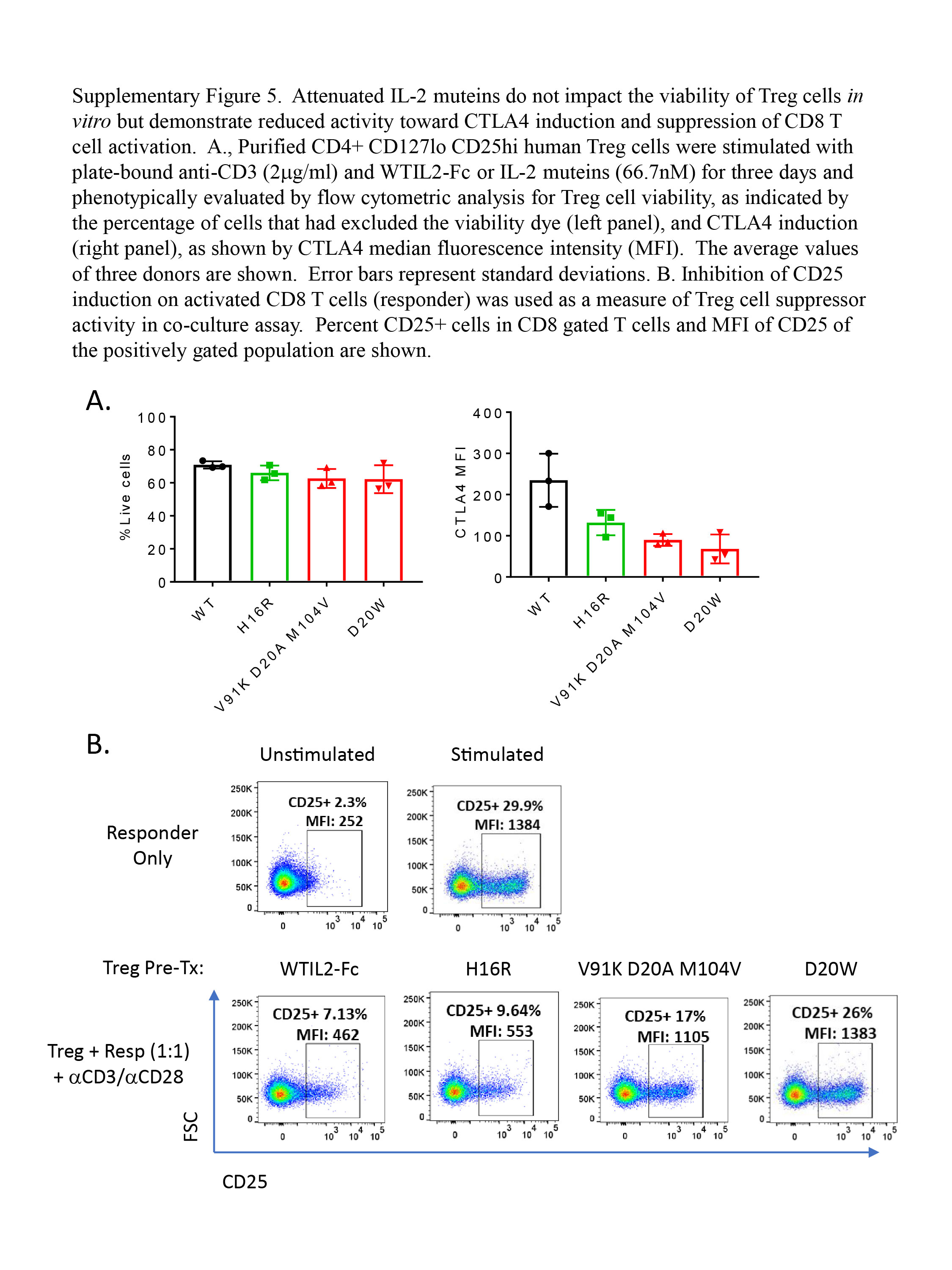

Supplement: Supplementary file 5 [file Image_5.jpg]

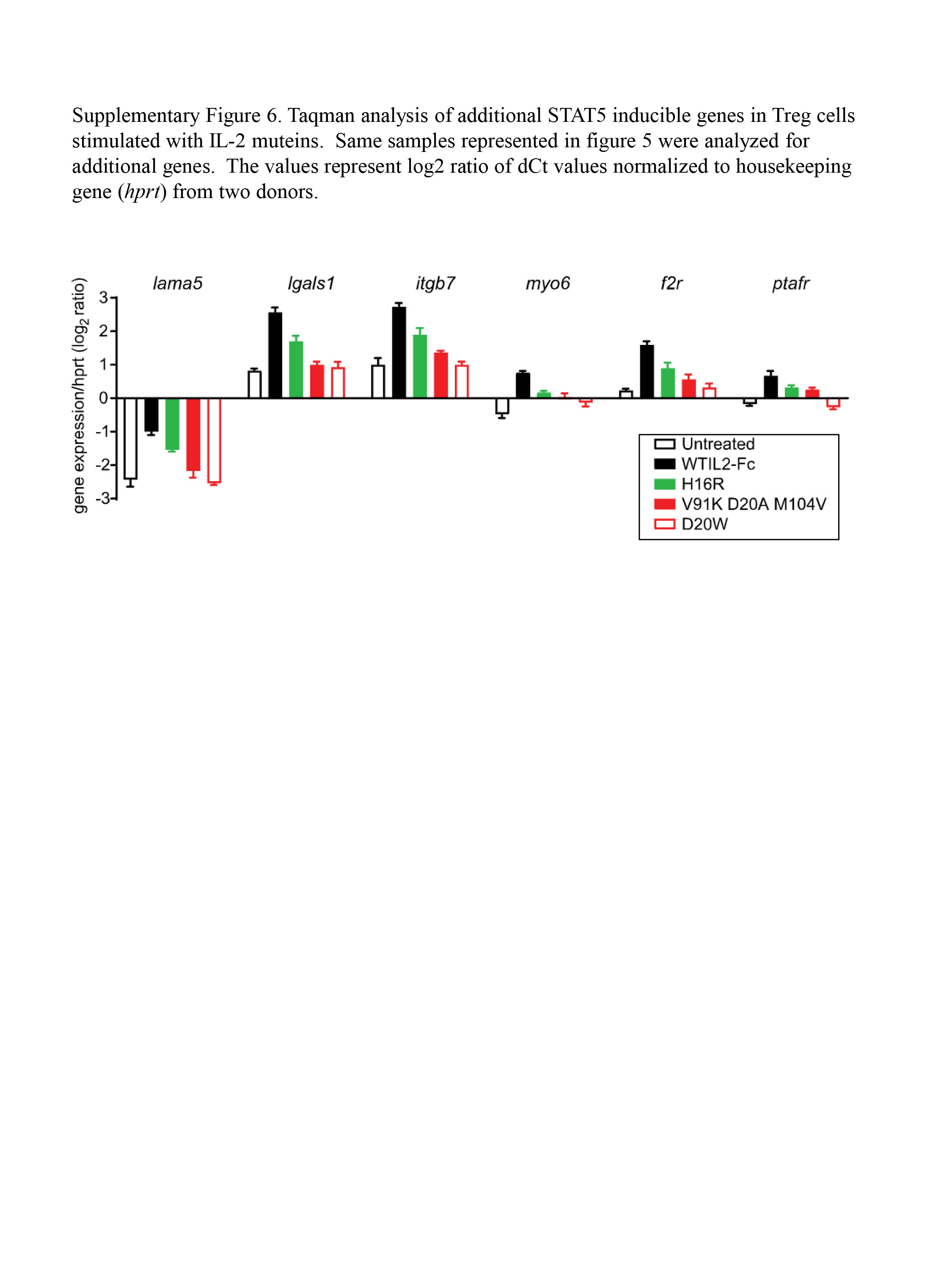

Supplement: Supplementary file 6 [file Image_6.JPEG]

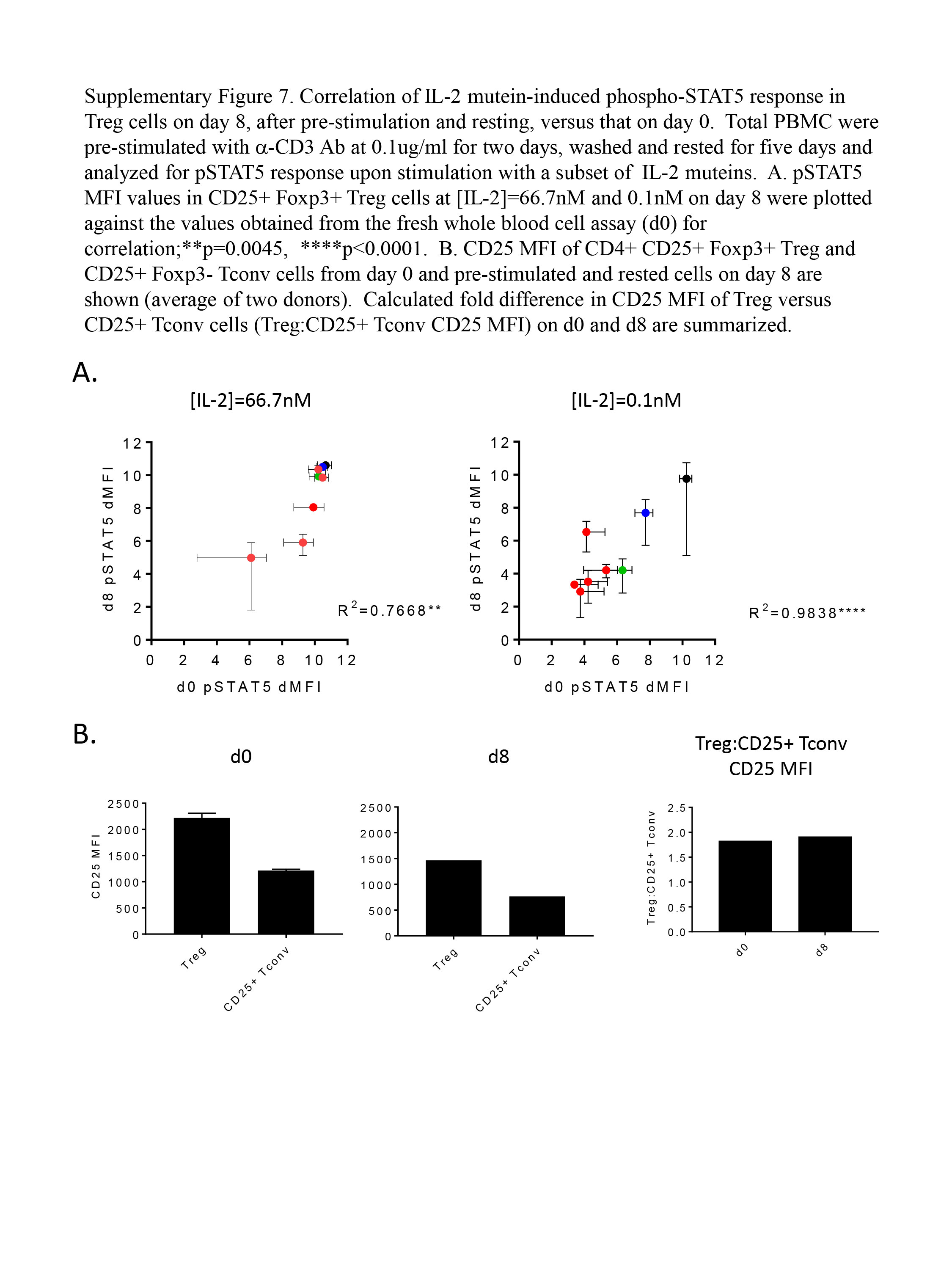

Supplement: Supplementary file 7 [file Image_7.jpg]

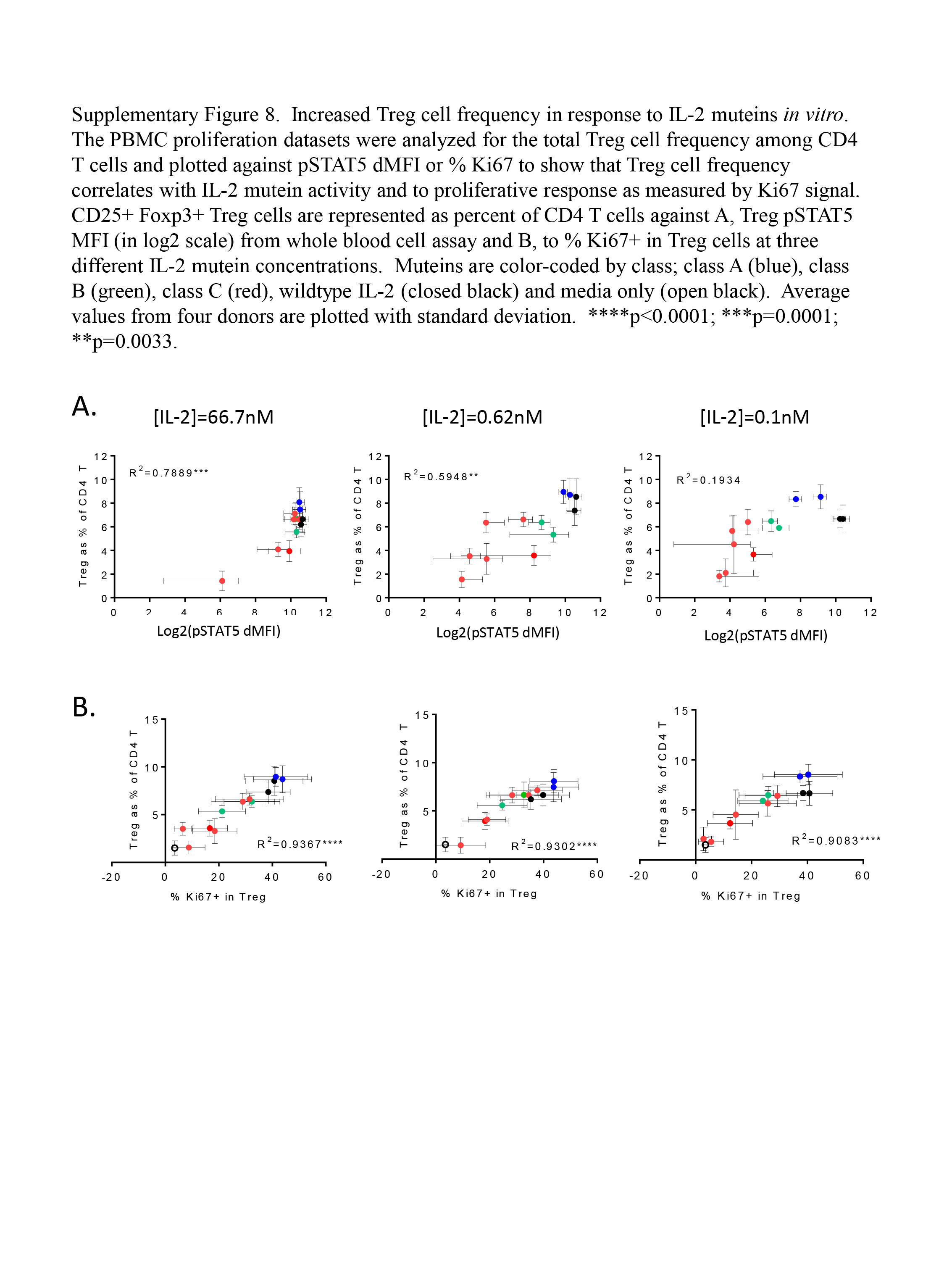

Supplement: Supplementary file 8 [file Image_8.JPEG]
